# Context-specific life cycle emissions pathways for EU buildings and construction

**DOI:** 10.1038/s41467-026-73433-1

**Published:** 2026-05-25

**Authors:** Nicolas Alaux, Nicolas Bechstedt, Xiaoyang Zhong, Alessio Mastrucci, Delphine Ramon, Dominik Steinberger-Maierhofer, Karen Allacker, Alexander Passer, Martin Röck

**Affiliations:** 1 Graz University of Technology, Institute of Structural Design, Working Group Sustainable Construction, Graz, Austria; 2 Energy, Climate, and Environment (ECE) Program, International Institute for Applied Systems Analysis (IIASA), Laxenburg, Austria; 3 Tsinghua University, Tsinghua Shenzhen International Graduate School, Institute of Environment and Ecology, Shenzhen, China; 4 KU Leuven, Faculty of Engineering Science, Department of Architecture, Leuven, Belgium; 5 RISE Institute for Regenerative Spatial Systems Science, Vienna, Austria

**Keywords:** Climate-change mitigation, Climate-change policy, Socioeconomic scenarios, Climate-change mitigation

## Abstract

The European Union aims to reduce greenhouse gas emissions by 55 percent by 2030 relative to 1990 and achieve climate neutrality by 2050. Yet, translating these targets into pathways for buildings and construction is challenging across diverse national contexts. We model building stocks for the twenty-seven Member States of the European Union and evaluate 4096 life cycle emissions scenarios, considering national capacities. Here we show that, over 2020–2050, achieving these targets would require avoiding 8.53 billion metric tons of carbon dioxide equivalent, approximately ten years of emissions at 2020 levels. Mostly relying on improving energy efficiency and material production would exceed national capacities by 2.72-billion-ton, 32 percent of the required reduction. A combined approach that also reduces per capita space demand, applies circularity measures, and uses bio‑based materials could achieve an additional 2.19 billion tons within national capacities. For each country, we identify the strategies that maximize projected reductions to inform policy design.

## Abstract (web summary)

The study identifies feasible, country-specific pathways for EU buildings to meet the climate targets, avoiding 8.5 billion tons CO_2_e. Success needs more than energy efficiency: it also needs less space per person, circularity, and bio-based options.

## Introduction

The built environment is a critical driver of climate change, with the construction and operation of buildings accounting for 21 % of global greenhouse gas (GHG) emissions^1^ and 37 % of energy- and process-related carbon dioxide (CO_2_) emissions^2^. Life cycle emissions of buildings include both operational emissions, generated during the energy use, and embodied emissions from materials production, transport, construction, and end-of-life processes. Historically, policy has primarily targeted operational emissions, leading to significant energy efficiency gains in new buildings. This progress has elevated the relative importance of embodied emissions, which now constitutes a major, and increasingly scrutinized, share of a building’s life cycle emissions^3^. This shift underscores the necessity of adopting a whole life cycle perspective to accurately assess environmental impacts and formulate comprehensive mitigation strategies for buildings and construction^4^.

Translating the European Union’s (EU) climate targets, including a 55 % reduction in GHG emissions by 2030 compared to 1990 levels and climate neutrality by 2050, into actionable pathways for the building stock remains challenging. Building stock models provide a scientific foundation to evaluate policy impacts and guide decision-making^5,6^, and there is ongoing need for high-quality, large-scale data^7^. At the supranational scale, there are bodies of work addressing material‑related embodied emissions^8,9^, and operational energy use^10–13^. Integrating these dimensions within a single framework requires harmonized data and methods, and limited spatial resolution can hinder the representation of diverse national contexts and capacities.

Building stock models are used to explore the landscape of GHG mitigation strategies, including circularity measures, per capita space demand reduction, bio‑based materials use, operational emissions reduction, material production improvement, as well as transport and construction processes improvement^14^. Translating these strategies into practice across heterogeneous EU Member States depends on national implementation capacity and context: resource and industrial conditions (e.g., availability of bio‑based materials, recycling rates), geographic and building stock characteristics (e.g., renovation potential, seismic stability), and socio‑economic and policy landscapes (e.g., regulatory frameworks, workforce skills, social conditions). These factors shape the realism of projections at supranational scale. Context is particularly relevant for sufficiency or demand‑side strategies, such as reducing average living area per capita, which aim to lower energy and material demand. Given their potential for early adoption and relatively low economic or technical barriers, such measures can deliver meaningful near‑term GHG emission reductions while capital‑intensive, low‑carbon technologies continue to mature and scale^15^.

In this work, we quantify the life cycle GHG emissions for the EU-27 building stock over 2020–2050 using PULSE-EU (Prospective Upscaling of Life cycle Scenarios and Environmental impacts for EU buildings)^16^, a bottom-up, archetype-based model that integrates life cycle assessment from the combined MMG (Environmental Profile of Building Elements) and SLiCE (Scalable, high-definition Life Cycle Engineering) models^17,18^ with material flow analysis at Member State (MS) level. We integrate a set of 15,026 building archetypes representing both residential and non-residential buildings, for which we build hierarchical inventories from whole building to components and materials using established datasets (e.g., AmBIENCe^19^, Hotmaps^20^, Building Stock Observatory^21^). We validate against the MESSAGEix‑Buildings (Model for Energy Supply Strategy Alternatives and their General Environmental Impact) and STURM (Building Stock Turnover Model) independent models^22,23^ and implement measure‑driven scenarios that operationalize six strategy groups (circularity, reduced space demand, bio‑based materials, operational emissions reduction, improved material production, and improved transport/construction) at low, medium and high ambition, with national implementation capacity explicitly embedded. An online whole life carbon (WLC) scenario explorer further enables activation of individual strategies and assessment of alignment with EU targets^24^. Full methodological details are provided in Methods and the Supplementary Information.

## Results

### Emission futures for EU buildings and construction

**Fig. 1:**
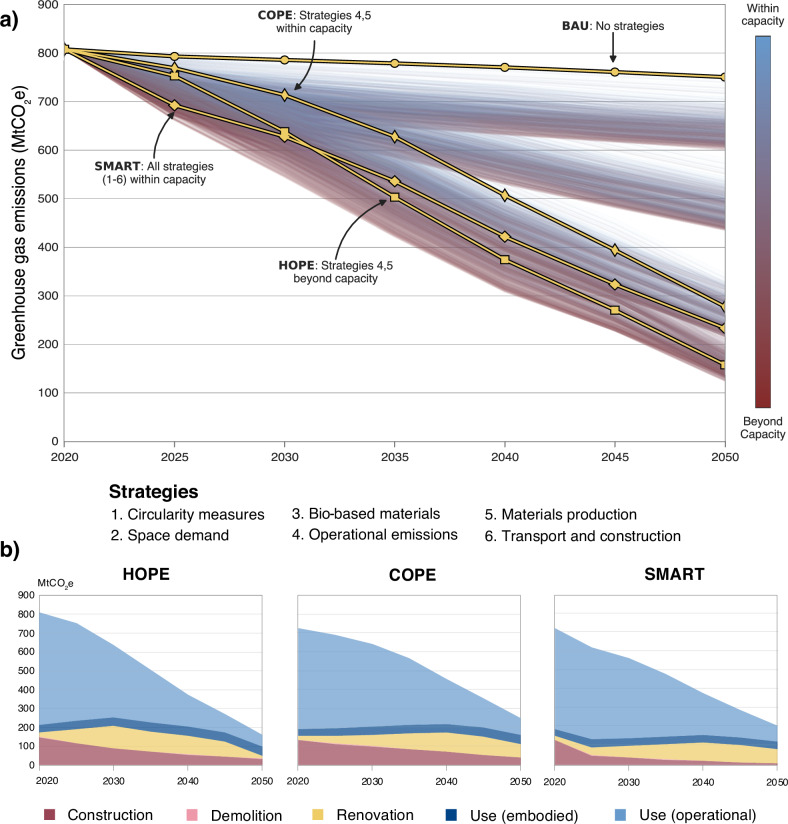
This figure displays annual life cycle greenhouse gas (GHG) emissions for the building stock of the European Union (EU) across multiple future scenarios, detailing strategy implementation and showing emission breakdowns by life cycle stage for three key scenarios. It is divided into: **a** Annual life cycle GHG emission results of future scenarios for the EU building stock, including all 27 Member States (MS). The main policy-relevant scenarios, which include the BAU (Business-As-Usual), HOPE (Honoring Official Policy Expectations), COPE (Capacity-Orchestrated Policy Execution) and SMART (Strategy Mix Approach for Robust Trajectories) scenarios, are shown as thicker lines on the graph. The faded lines in the background represent 4096 additional scenarios which were generated to explore the solution space of potential futures (as explained in Supplementary Note 1), which can be activated individually using the online whole life carbon scenario explorer^24^. These scenarios are colored depending on the implementation of the strategies, ranging from blue (implemented within MS capacity) to red (implemented beyond MS capacity). **b** Annual GHG emissions of the HOPE, COPE and SMART scenarios divided per life cycle stage, showing their different building stock dynamics. These results are based on the upscaling of building archetype data to the EU building stock and the projection of future scenarios. This figure was generated with python using matplotlib (v3.10.8) and polars (v1.39.3).

EU buildings and construction (excluding infrastructure) emitted 808 MtCO_2_e in 2020 (including fossil, biogenic, land use and land use change emissions) due to their construction (18 %), maintenance (5 %), operation (73 %), renovation (3 %) and demolition (1 %). These results are based on the upscaling of building archetype data to establish a baseline year in 2020 for the EU building stock (see Methods). Future GHG emissions strongly depend on the strategies implemented by the different MS and their level of implementation (see Table 1 or Supplementary Note 1). While the Business-As-Usual (BAU) scenario is projecting current trends, the HOPE (Honoring Official Policy Expectations) scenario meets official policy targets but national capacities are exceeded. Its more realistic counterpart COPE (Capacity-Orchestrated Policy Execution) scenario respects these capacity limits, but does not meet all policy targets, and the SMART (Strategy Mix Approach for Robust Trajectories) scenario uses an extended strategy mix to meet targets within those capacities. Resulting emissions in 2050 are ranging from 751 million metric tons of carbon dioxide equivalent (MtCO_2_e) (BAU) to 158 MtCO_2_e (HOPE) (Fig. 1a).

**Table 1:** Overview of the policy-relevant scenarios applied to the building stock of the European Union (EU)

Scenario	Short description	Strategies scope	Policy targets	Member State capacity
BAU	Business-As-Usual: A theoretical reference scenario which is a projection of the baseline year activity rates.	None: no further action.	Not reached.	Satisfied.
HOPE	Honoring Official Policy Expectations: Assuming current policies are fully delivering, and policy targets are being met as planned using the strategies expressed in current policy documents.	Limited: Mainly the reduction of operational emissions and improvement of material production processes, and to a smaller extent, the improvement of transport and construction processes, as well as circularity measures (reuse and recycling).	Strictly reached (Emissions Trading System, Energy Efficiency Directive, Renewable Energy Directive, Energy Performance of Buildings Directive and Waste Framework Directive).	Exceeded.
COPE	Capacity-Orchestrated Policy Execution: Uses the same strategies as expressed in current policy documents but their implementation is limited by Member State capacity.	Limited: Mainly the reduction of operational emissions and improvement of material production processes, and to a smaller extent, the improvement of transport and construction processes, as well as circularity measures (reuse and recycling).	Partially reached (only Energy Performance of Buildings Directive, Renewable Energy Directive and Waste Framework Directive)	Satisfied.
SMART	Strategy Mix Approach for Robust Trajectories: Goes beyond the strategies expressed in policy documents to reach the policy targets.	Extended: Implementation of circularity measures, reduction of per-capita space demand, shift to low carbon, bio-based materials, reduction of operational emissions, improvement of material production, transport and construction processes.	Implicitly reached (emissions in 2030 for which the targets are specified are lower than in the HOPE scenario).	Satisfied.

It includes a short description of the scenario, the name of the strategies activated to generate the scenario, the EU policy targets reached and whether Member State capacity was exceeded. More details regarding the modeling of these scenarios are available in Supplementary Note 1.

Assuming that the targets formulated in current EU policies, such as the Energy Performance of Buildings Directive (EPBD), are fully reached, by implementing the strategies expressed in these policy documents (HOPE, see Supplementary Table 7), we find that the contribution of buildings and construction to achieving the EU climate targets would imply a cumulative GHG emissions reduction of 8.53 billion metric tons of carbon dioxide equivalent (GtCO_2_e) from 2020 to 2050. This represents approximately a full decade of their baseline year emissions. As current EU policies mainly focus on the reduction of operational emissions and improvement of material production processes, reaching policy targets with these two strategies requires going beyond national implementation capacities (Fig. 1a). Limiting the use of these strategies to MS capacity (COPE) comparatively leads to an increase in cumulative emissions of 2.72 GtCO_2_e (32 % of the required reduction), highlighting the gap between theoretical mitigation potential and practical implementation capacities. This presents a risk to the achievement of EU climate targets. However, a large share of this increase in emissions (2.19 GtCO_2_e or 80 %) could be compensated by activating a wider range of strategies beyond the ones expressed in policy documents (SMART), while still operating within realistic MS capacities (Supplementary Fig. 6), as the result of our analysis demonstrates. This offers a more balanced and secure approach, while achieving similar cumulative GHG emissions.

These scenarios furthermore reveal synergies between strategies and timing of implementation (Fig. 1b). The HOPE scenario models an accelerated renovation wave, to strictly fulfill energy efficiency policy targets (supplementary Tables 7 and 9) peaking at 3.6 % in 2030 and staying above 3 % until 2040, which results in a rapid decline in operational emissions. In contrast, the COPE scenario, which incorporates plausible implementation capacities, for which the list of criteria is provided in Supplementary Table 5, projects a delayed peak in renovation activity to 3 % in 2040, while only reaching 2 % in 2030. The SMART scenario demonstrates that this deferred mitigation can be effectively counteracted through circularity measures, reduction of per capita space demand and increased use of bio-based materials. These measures reduce the overall GHG emissions by minimizing the demand for new construction while concurrently decreasing operational energy demand through more efficient use of the existing building stock.

### EU climate policy in light of national contexts and capacities

**Fig. 2:**
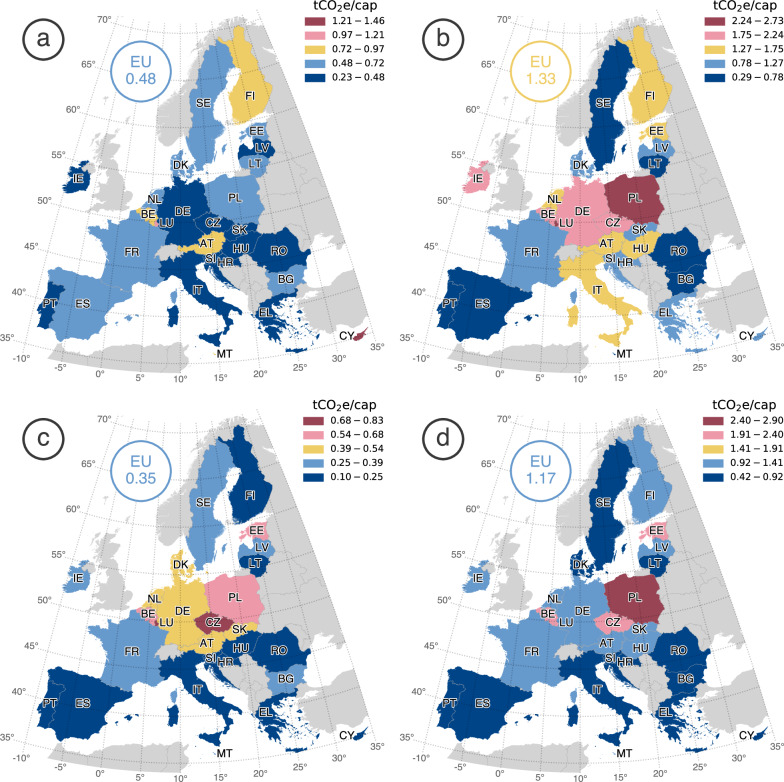
This figure presents per capita embodied and operational greenhouse gas (GHG) emissions for Member States (MS) of the European Union (EU) in 2020, and the projected per capita life cycle GHG emission reductions by 2030 and 2050 under the SMART (Strategy Mix Approach for Robust Trajectories) scenario compared to BAU (Business-As-Usual), highlighting potential shared reduction efforts. It is divided into: **a** Embodied GHG emissions per capita for each individual MS in 2020 (in tons of carbon dioxide equivalent per capita, or tCO_2_e/cap). **b** Operational GHG emissions per capita for each individual MS in 2020 (in tCO_2_e/cap). **c** Reduction in life cycle GHG emissions between SMART and BAU for 2030, per capita (in tCO_2_e/cap). **d** Reduction in life cycle GHG emissions between SMART and BAU for 2050, per capita (in tCO_2_e/cap). **C**, **d** were obtained by subtracting the GHG emissions of the BAU scenario from the ones of the SMART scenario (in 2030 or 2050), and then dividing by the population in the respective year. This indicates how the GHG emission reduction effort can be shared among EU MS in the SMART scenario. In all graphs, the value in the circle where EU is written represents the average value across all EU MS. Country abbreviations use EU codes per the Interinstitutional Style Guide. Underlying data for this figure is available in Supplementary Table 3. This figure was generated with python using matplotlib (v3.10.8), shapely (v2.1.2), pandas (v3.0.2), openpyxl (v3.1.5), geopandas (v1.1.3) and geos (v3.14.1), using the administrative areas from GADM (v2.8).

The success of EU climate policy is challenged by the diverse contexts and building stocks across MS. Embodied GHG emissions per capita in 2020 range from 0.23 tons of carbon dioxide equivalent per capita (tCO_2_e/cap) in Greece and Italy to 1.46 tCO_2_e/cap in Cyprus, with an average of 0.48 tCO_2_e/cap at the EU level (Fig. 2a). Smaller countries, such as Cyprus, Luxembourg, Malta, and others (Austria and Belgium), have higher construction activity on a per capita basis compared to other countries, reflected in higher embodied emissions. By contrast, several Southern (Greece, Italy) and Central‑Eastern (Romania, Slovakia, the Czech Republic, Croatia or Hungary) MS exhibit relatively low per‑capita embodied emissions.

Operational GHG emissions range from 0.29 tCO_2_e/cap in Sweden to 2.73 tCO_2_e/cap in Luxembourg, with an EU average of 1.33 tCO_2_e/cap (Fig. 2b). These emissions follow different patterns than the embodied emissions, with Luxembourg, Poland, the Czech Republic, Ireland and Belgium having the highest emissions per capita, indicating a higher reliance on fossil-fuel based heating systems and a colder climate. Conversely, Sweden, Portugal, Bulgaria, Spain, Lithuania, Malta and Romania have the lowest operational emissions per capita. For countries like Sweden, which also have a cold climate, this reflects higher energy efficiency of the buildings and a low-carbon energy mix. For Southern European countries (Spain, Portugal), although space cooling is higher than in other countries, the relative increase remains smaller than the decrease in space heating, ensuring overall low operational emissions per capita.

Looking at the per capita emission reduction requirements for 2030 (Fig. 2c), regional patterns emerge. Western Europe shows high requirements in Luxembourg, Belgium, the Netherlands, Austria, Germany and Denmark (all above 0.40 tCO_2_e/cap) indicating industrialized economies with higher implementation capacity, but also higher per capita operational emissions in 2020 (e.g. Luxembourg, Belgium, Germany). Southern European countries have consistently lower requirements (Spain, Portugal, Greece stay below 0.15 tCO_2_e/cap), echoing their lower per capita life cycle emissions in 2020. Nordic Countries demonstrate moderate requirements (around 0.25 tCO_2_e/cap for Sweden and Finland) despite high living standards, reflecting their low-carbon energy mixes. Eastern Europe is highly variable, with some countries (Czech Republic, Estonia, Poland) facing steep requirements due to higher per capita emissions in 2020, while others (Hungary, Romania, Lithuania) have more modest targets.

In 2050 (Fig. 2d), while Poland, Estonia, Luxembourg, Belgium and the Czech Republic keep their high emission mitigation requirements, Southern European countries maintain the lowest requirements. Nations like Greece (5.7x increase), Romania (5.2x), and Poland (5.1x) experience rapid acceleration compared to Nordic countries with more gradual progressions. The substantial variation in per capita emission reduction requirements, ranging from 0.10 to 0.83 tCO_2_e/cap in 2030 and 0.42 to 2.90 tCO_2_e/cap in 2050, combined with the heterogeneous acceleration patterns across MS, indicate that effective climate policy must incorporate differentiated approaches that account for national constraints and implementation capacities. These include, as provided in Supplementary Table 5, resource and industrial capacity (e.g., bio-based material availability, recycling rates), geographic and building stock characteristics (e.g., renovation potential, seismic stability), and the prevailing socio-economic and policy landscapes (e.g., national policy frameworks, workforce skills, social conditions)^14^.

### Extending the EU policy focus for context-specific mitigation pathways

**Fig. 3:**
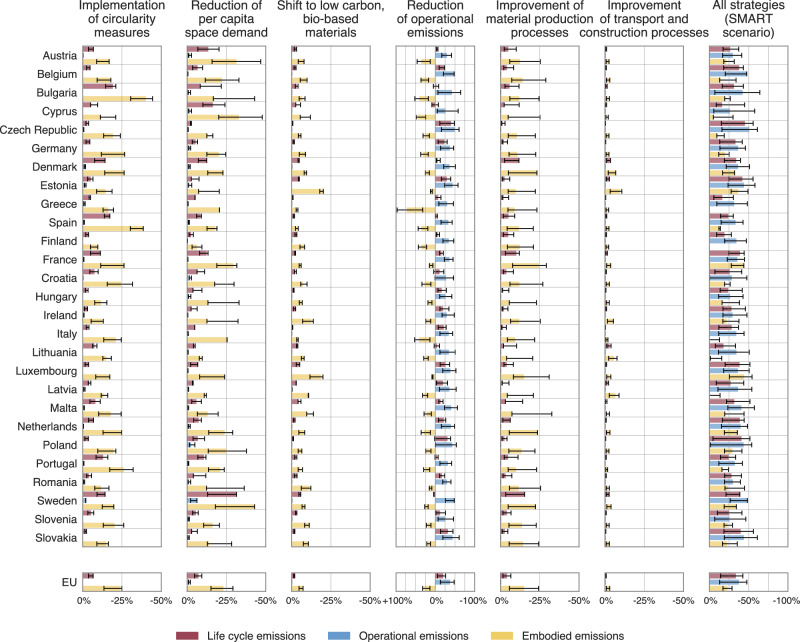
This figure presents the relative change in cumulative greenhouse gas (GHG) emissions when applying a specific strategy, or all of them in the case of the SMART (Strategy Mix Approach for Robust Trajectories) scenario, compared to the BAU (Business-As-Usual) scenario, reported in percent (%). More specifically, the annual GHG emissions over the 2020 to 2050 time period were summed for both the BAU scenario and a scenario in which the strategy is implemented to the capacity of each Member State (MS), and the relative difference between these cumulative emissions was computed. A negative value means a reduction in emissions for the scenario in which the strategy is implemented, compared to the BAU. The whiskers represent the sensitivity intervals regarding the implementation capacity of each MS (given by generating the results with a low and a high implementation potential). The bar represents the results with the actual implementation potential (e.g. low, medium or high). Note that the scale of the x‑axis caps at 50 % for strategies that do not exceed this threshold. Underlying data is available in Supplementary Table 8. This figure was generated with python using matplotlib (v3.10.8) and polars (v1.39.3).

EU policy provides a solid framework for reducing GHG emission that target some of the most effective strategies, namely the reduction of operational emissions, especially via the energy efficiency targets of the EPBD and the Energy Efficiency Directive (EED), as well as the improvement of material production processes, with the emission reduction targets from the Emissions Trading System (ETS). However, the analysis of strategy effectiveness across 27 MS reveals significant heterogeneity (Fig. 3). Operational emission improvements include thermal renovations, exchanges in heating systems, an increased share of renewable energy in the electricity and district heating, as well as the reduction in temperature setpoints. Though most effective at the EU level, they only represent the optimal strategy for 19 MS (when considering life cycle emissions). In particular, the Czech Republic achieves the highest reduction (40 %) due to elevated per capita operational emissions and a limited embodied emissions trade-off.

Building upon this EU framework, national authorities could complement their current strategy packages with the most effective ones for their country. In particular, three distinct groups emerge among countries where operational improvements are less effective: (i) High embodied emission countries per capita (e.g., Austria, Cyprus) with high construction rates benefit most from reductions in the per capita space demand, which can rapidly reduce the number of new constructions through better use of the available, existing building stock, while the reduction of operational emissions is slower to implement, with a large trade-off in embodied emissions; (ii) Low-carbon energy mix countries with high living standards (e.g. Sweden) also favor reductions in per capita space demand, because these embodied emissions dominate their life cycle emissions while other strategies, such as reduction in operational emissions or the improvement of material production processes, might be less effective than in other countries, due to their current use of low-carbon energy; (iii) Countries with balanced operational-to-embodied emission ratios (higher than 40–50 %, including Portugal, Spain, Bulgaria, Denmark, Lithuania) achieve optimal results through circularity measures, which include the extension of the service life of buildings through renovation, vacancy reduction, efficient structural material use, as well as increased reuse and recycling. This reflects the smaller influence of operational emissions and could indicate higher vacancy rates, demolition rates or a higher potential for reuse and recycling. The complementary nature of these strategies also stands out, as combined implementation consistently outperforms individual approaches. When aligned with MS capacity, integrated strategies could reduce cumulative life cycle emissions by 34 % EU-wide.

Focusing on embodied GHG emissions also reveals clear national priorities: circularity measures show high potential in Bulgaria and Spain, the reduction of per capita space demand in Austria and Cyprus, while bio-based materials offer greater benefits in Estonia. Some countries (Romania, Ireland) demonstrate similar effectiveness (12–13 % reduction) across multiple strategies, while others (Netherlands) achieve comparable results (24–25 %) through circularity, reduction of per capita space demand, or improvements of material production processes. While current EU policy targets mostly rely on the improvement of material production processes to reduce embodied emissions, this strategy is rarely the most effective one from an embodied emissions perspective, revealing an untapped potential for future embodied emissions mitigation policies, which could be overtaken in national policies to complement current EU targets.

## Discussion

Our analysis indicates that current EU policy targets, which predominantly focus on reducing operational GHG emissions, and improving the production of construction materials, may surpass the implementation capacity of numerous MS, thereby jeopardizing the attainment of climate goals. Across the EU, our results show a clear prioritization: (i) reducing operational emissions is the most effective strategy overall; (ii) reducing per capita space demand is next‑best in MS with high embodied emissions per capita or low‑carbon energy mixes; (iii) circularity measures are most effective where operational and embodied emission shares are similar (40–50 %); (iv) improvements in material production processes deliver smaller gains on average; and (v) shifting to low‑carbon, bio‑based materials and improving transport and construction processes typically have the lowest EU‑wide impact, albeit with country‑specific exceptions (e.g., stronger benefits from bio‑based materials in Estonia).

This hierarchy, together with capacity constraints, is consistent with previous research suggesting that highly ambitious, extreme scenarios often lack practical viability^25^, as societal and systemic changes tend to converge towards more moderate pathways^26^. In the specific context of building renovation, it has likewise been established that a uniform, EU-wide strategy is not necessarily optimal from a cost-benefit perspective for reducing operational emissions, and that more targeted approaches tailored to national contexts are advisable^27^. Our study provides a quantitative framework for differentiating GHG emission reduction efforts among MS, identifying the most effective and feasible mitigation strategies for each national context. Because EU law prevails but MS retain discretion when transposing EU directives, our framework shows how to use this discretionary space to tailor measures and improve the effectiveness of the EU regulatory framework at national level.

Reducing operational GHG emissions being the most effective lever to reduce life cycle emissions in most MS, national policies should translate the EPBD, EED, Renewable Energy Directive (RED), and the forthcoming ETS for buildings and road transport (ETS2) into a coherent package of actions. ETS2 will introduce an upstream carbon price on fossil heating fuels (planned from 2027), which, together with complementary national policies, can reinforce performance standards and targeted support by strengthening incentives for fuel switching and reducing energy demand.

Key additional measures from the EPBD include adopting comprehensive National Building Renovation Plans that chart a pathway to a climate neutral stock by 2050, enforcing Minimum Energy Performance Standards for non‑residential buildings, and applying the Zero Emission Building standard to all new construction while progressively incorporating whole life carbon limits. Supporting instruments, such as Energy Performance Certificates, Renovation Passports, and targeted financial incentives must be widely available^28^. The EED’s Energy Efficiency First principle should guide planning, investment and permitting, with the public sector renovating at least 3 % of its floor area annually and prioritizing deep renovation measures. To ensure affordability and implementation capacity, MS should earmark a share of ETS2 auction revenues and leverage the Social Climate Fund (2026–2032) to co‑finance deep renovations, including exchanges of heating systems, while providing temporary, targeted income support for vulnerable households and micro‑enterprises. In parallel, the RED promotes the accelerated deployment of on-site renewable generation and establishes enabling frameworks for energy communities^29^. These policies must be complemented by measures that mitigate embodied emissions, and allow strategies to be deployed where they are most effective, thereby navigating implementation capacity constraints.

Sufficiency measures, which aim to reduce the absolute demand for energy and materials to operate within planetary boundaries, can induce profound systemic effects^30,31^. In our study, they include the reduction of per capita space demand (most effective in in Austria and Cyprus), the extension of the service life of buildings through renovation and vacancy reduction (included in the circularity measures, most effective in Portugal, Spain, Bulgaria, Denmark and Lithuania), as well as the reduction in temperature setpoints (part of the operational emission measures). Despite being underrepresented in policy compared to efficiency and renewable energy strategies^32^, their importance is well-established. Previous studies have shown that sufficiency is crucial for achieving carbon neutrality and energy security in Europe, as it alleviates pressure on supply-side transformations^33^. Indeed, scenarios that exclude behavioral change are projected to miss climate neutrality targets in the EU^34^. Furthermore, from an energy systems perspective, integrating sufficiency is highly advantageous, as a lower electricity demand reduces the need for costly supply-side investments and grid infrastructure^10^. Our results corroborate these findings, demonstrating that incorporating sufficiency, alongside with other measures, creates a more balanced and feasible GHG emissions trajectory.

Sufficiency‑oriented policies should prioritize the optimal use of the existing building stock to avoid upfront embodied GHG emissions associated with new construction, while simultaneously reducing operational emissions through higher occupancy and intensified use of floor space. Effective implementation requires coordinated measures, such as establishing vacancy registers and applying vacancy taxes to incentivize the re‑entry of under‑occupied units into the market^35^, streamlining permitting procedures for repurposing and retrofits, and offering density bonuses or zoning concessions to reward adaptive reuse^29^. Other examples include raising awareness, rethinking communication and designing effective financial motivation, such as a combination of different financial incentives that reward downsizing through flat swaps, or the introduction of minimum occupancy rates^36^. Public authorities and developers can further stimulate flexible, resilient design by launching competitions and pilot projects focused on adaptable concepts, and by creating neighborhood‑level agencies that advise tenants on downsizing or relocation across life stages.

However, the practical application of sufficiency faces considerable challenges, notably socio-political acceptance and potential rebound effects^37^, necessitating fundamental reforms in both the content and the design process of building sector policies^30^. Measures perceived as limiting choice or comfort, such as minimum occupancy thresholds, downsizing incentives, or lower temperature set‑points, often face stronger opposition than efficiency standards^36,38^. Instruments like vacancy registers and taxes also require significant administrative capacity and proportional enforcement. Cultural norms around dwelling size, privacy and thermal comfort can limit voluntary uptake or lead to rebound effects. Without careful design and clear exemptions, they may trigger privacy and property‑rights concerns or legal challenge. Examples of enablers that could raise acceptability and uptake are comfort guarantees and building‑level controls when optimizing temperature set‑points, to avoid substitution with portable heaters, or tenant protections and anti‑displacement measures when promoting downsizing or intensified use, which could be in the form of temporary rent caps or indexation limits for participants. Evidence additionally indicates that acceptance improves when policies are co‑designed with residents and social partners, distribute costs visibly and fairly, preserve choice rather than introduce bans, and deliver clear co‑benefits (health, comfort, affordability)^39,40^.

For countries showing high potential to reduce embodied GHG emissions by using bio-based materials or improving materials production processes, as well as reuse and recycling, such as the Estonia, Romania, Ireland or the Netherlands, policy measures should combine incentives, standards, and market‑creation mechanisms. Governments can introduce tax credits or subsidies for the manufacturing and procurement of certified bio-based products, while mandating minimum bio‑content thresholds in public‑sector contracts. In parallel, maintaining and enhancing the carbon sink capacities in land-based systems that provide these bio-based products is crucial^41^. Updating building codes and material specifications to recognize and reward low‑embodied emissions bio-based materials encourages their adoption in construction. In parallel, standards for circularity, such as material passports, product‑level environmental data, and verified recycled‑content labels, facilitate traceability and market confidence^29^. In parallel, implementing extended producer responsibility schemes, take‑back obligations, and incentives creates economic drivers for systematic reuse, recycling, and urban mining of bio-based and conventional materials, closing material loops and lowering overall embodied emissions^29^.

The findings of this study prompt a critical re-evaluation of net-zero emission frameworks as applied to the building sector. Our analysis indicates that even highly ambitious GHG mitigation pathways leave a substantial residual emissions gap, which net-zero paradigms typically propose to address through Carbon Dioxide Removal (CDR). Considering the limited application of CDR in buildings^42^, the reliance on such technologies would introduce considerable uncertainty and risks diverting focus from the primary goal of maximizing direct emissions abatement, a priority underscored by the escalating climate crisis^43,44^. Furthermore, the integrity of net-zero calculations is challenged by methodological limitations in carbon accounting, particularly concerning biogenic materials. The accounting method used in this study, common in the field, omits the carbon opportunity cost of biomass harvesting^45^. Recent evidence suggests the resulting loss of forest sink capacity can be greater than the material substitution benefit^41^, implying that the net climate contribution of such strategies may be systematically overestimated. There is a clear need to develop integrated assessment models that couple building stock dynamics with land-use and ecosystem science. Such interdisciplinary approaches are essential for establishing a more comprehensive understanding of the carbon cycle and for ensuring that building sector mitigation strategies are genuinely effective.

Finally, although rooted in the EU, the study’s key insights (requiring a balanced strategy mix, emphasizing sufficiency to alleviate systemic stress, and acknowledging limited implementation capacity), could apply broadly to mature economies in the Global North with comparable building stock dynamics (e.g., North America, Australia). Consequently, the work provides a transferable framework for national emission mitigation pathways, while noting that further research is needed for regions with scarce data, weak institutional capacity, or extensive informal settlements.

## Methods

### Building stock characterization and aggregation

The building stock is characterized using an archetype-based, bottom-up approach. Archetypes are typically developed through statistical analysis to reflect the diversity in building age, size, construction practices, equipment, and consequently emission profiles. They are virtual representations of various buildings in the stock that share similar characteristics. To define the EU building stock in this study, representative archetypes have been created at the MS level, clustering buildings based on sector (residential or non-residential), building typology (e.g., single-family house, office), and construction period (e.g., 1970–1979). Nine building typologies are modeled, including residential (single-family houses, multi-family houses, and apartment blocks) and non-residential (offices, trade, education, health, hotels and restaurants, and other non-residential buildings). The classification used for these building archetypes aligns with the classification used in the Building Stock Observatory (BSO)^21^, the most up-to-date framework in the EU. Three categories of archetypes are created: one for existing buildings, one for new buildings and one for thermal renovation of existing buildings. An overview of the number of building archetypes for each category is provided in Supplementary Table 1.

For existing buildings, the stock is represented by 66 archetypes per EU MS, resulting in a total of 1782 archetypes. In addition, variants in technical systems are included, leading to 4490 archetypes. Each archetype is then assigned information regarding its building geometry, material composition, as well as heating, ventilation and air conditioning systems (HVAC), called attributes. Attributes relative to building geometry are first derived from AmBIENCe^19^, then complemented with data from the Cost-effectiveness studies^46^ and TABULA/EPISCOPE^47^. Regarding material composition, Hotmaps^20^ is used to derive the materials composing floors, walls, roofs and windows, and the construction technologies are then detailed in terms of layer thickness and performance properties using AmBIENCe^19^. In order to validate the retrieved information and fill data gaps, a data review by regional experts who were part of the EU-WLC project^48^ was conducted. Finally, all attributes related to HVAC were derived from AmBIENCe^19^. The existing building stock is characterized by its total useful floor area, which is taken from the BSO^21^, alongside with building stock occupancy data, for different building archetypes (shown Supplementary Figs. 1 and 2).

New building archetypes are used for modeling new construction activity on building stock level. New archetypes are defined for all nine building typologies and all EU MS. A first set of 243 conventional new building archetypes is created assuming the same attributes for building geometry and material composition as the latest building age class (2011–2019). For each of the 243 new archetypes, three energy performance standards are specified. The first corresponds to the standard energy performance level, aligned with national nearly-zero energy building requirements as implemented by 2021. The second reflects an advanced energy performance, based on passive energy standards as defined per region in a recent study^49^. The third represents sub-standard performance, consistent with the energy characteristics of the latest building age class (2011–2019). HVAC systems are adapted to meet the energy performance requirements applicable in each MS, by means of adjustments to the system efficiency and the phase-out of gas- and oil-based space heating systems in new buildings. This was validated by regional experts who were part of the EU-WLC project^48^. Possible phase-out of gas- and oil-based space heating systems in each MS at the stock level depends on the projected scenarios. In total 1973 archetypes for new buildings are defined with a conventional construction material, which are complemented with timber archetypes (mass or lightweight timber construction with bio-based insulation)^50^, leading to 3946 additional archetypes for new buildings constructed in bio-based materials.

In addition, 4617 archetypes are generated for modeling renovation activities of different intensity. The modeling of renovation archetypes utilizes data from both existing building archetypes (for modeling the energy consumption), as well as new building archetypes (for the material composition of the respective renovation activity). First, building on the existing building archetypes, the energy performance levels of buildings in the stock are categorized and renovation options are modeled with the following levels of reduction in final energy consumption: Light renovation (expected energy savings <30 %, reduction factor of 0.25 applied); medium renovation (expected energy savings 30-60 %, reduction factor of 0.5 applied); deep renovation (expected energy savings >60 %, reduction factor of 0.75 applied)^51^. Depending on the existing building energy performance and the renovation depth (light, medium, deep), the scope of the intervention varies from a replacement and upgrade of insulation in external walls and roofs (light), to incorporating replacement and upgrade of windows, as well as, potentially, the replacement and upgrade of technical systems (medium, deep) that allows zero onsite operational emissions (heat pumps or district heating)^28^.

### Archetype inventory and life cycle assessment

For each of these archetypes, comprehensive life cycle inventories (LCI) are defined, with a hierarchical method for building decomposition^52^. More specifically, the modeling of buildings is structured in a hierarchical way from building archetype to building component (e.g. roof) to material level (e.g. concrete), using the material composition attributes that were defined for each archetype. These element definitions have undergone a review process by regional experts from the EU-WLC project^48^ to maximize the representation of the heterogeneity of existing buildings and to validate uncertain information. Life cycle impact assessment is conducted using the combined MMG (Environmental Profile of Building Elements)^18^ and SliCE (Scalable, high-definition Life Cycle Engineering)^17^ building life cycle model. Results are presented in this article with the global warming potential (GWP) total indicator using the Environmental Footprint 3.0 EN15804 + A2 method^53^. The modeling considers life cycle GHG emissions in line with EN 15978^54^ and following the latest EN 15804 + A2^55^ standards. It includes modules A1-A5 (for new building construction), B2, B4 and B6 (for buildings in use), B5 (for building renovation) and C1-C4 (for building demolition). Generic data from ecoinvent 3.6^56^ are used as background. To ensure geographical representativeness, for the production of materials, processes that are representative for the European market are chosen. National datasets are selected for the different building end-uses (e.g. electricity or gas burned in a boiler).

The building archetypes are assessed using a reference study period of 50 years, consistent with EU standards^28^. This period applies exclusively to the in-use emissions (B2, B4, B6). However, at the stock level, the service lives of the buildings are assessed differently, and it is common to have older than 50 years buildings still in use. To facilitate the upscaling to the building stock level, these in-use emissions are then annualized. These annual emissions are assumed to continuously happen as long as the building is in use, even beyond the 50 years reference period. The life cycle scenarios for transport, end-of-life waste treatments and reference service lives of building components are established at the MS level based on a literature review and expert consultation^57^. The B-PCR^58^ serves as a structure for these scenarios, in terms of building component and material classification. Scenarios for cleaning and maintenance during the use stage have been defined according to the Belgian MMG method^18^. The data and methodology behind the creation of these scenarios are openly available in a research data repository^59,60^.

The energy use for space heating and space cooling is based on the dynamic equivalent heating and cooling degree day methodology^61^ applied on building archetype level. In case a ventilation system is present in the building, electricity use is estimated based on the volume of the building and system efficiency in line with the EPBD’s calculation method. The energy use for domestic hot water is calculated based on the number of users for residential buildings and for non-residential buildings with a residential function^61^, and standard values are used for the other non-residential buildings^62^. The difference in operational GHG between EU MS is provided in Supplementary Fig. 5 in the case of apartment buildings, as an example.

### Spatial and temporal dynamics of the building stock

The upscaling of building archetype data to establish a baseline year in 2020 for the building stock (see Supplementary Figs. 3 and 4), along with the modeling of future scenarios for building stock development until 2050, is performed with the PULSE-EU (Prospective Upscaling of Life cycle Scenarios and Environmental impacts for EU buildings) building stock model (v1.0.0)^16^. This model is specifically developed to model the EU building stock, by expanding the foundational PULSE-AT model^63^, originally developed for Austria, thereby extending its scope to cover all 27 EU MS. PULSE-EU projects future building stock activities, including new construction, maintenance, renovation, replacement and demolition, following the equations and upscaling the logic from the Austrian model, with these main adaptations: (i) Austrian-specific data are replaced with EU-specific data (see Data Availability). (ii) The model is adapted to import files generated with the SLiCE^17^ building life cycle modeling structure, which affected the upscaling of in-use emissions (B2, B4, B6). (iii) The scenario modeling framework is adapted to be compatible with the input data and scalable to all EU-27 MS. (iv) The upscaling is performed based on useful floor area to align with the latest EPBD requirements^28^, which is slightly different from the net floor area used in the Austrian model.

New residential building constructions are first estimated based on the expected evolution of the national population and average living area per capita^64^. This results in an amount of area that is needed for housing every year. It is assumed that the average living area per person corresponds to the total useful floor area of buildings in use divided by the total population of this specific year. This might cause differences to other models or data sources, if the considered area is different, or if the vacant stock is included in this area. Second, the capacity of the building stock to provide housing is calculated. This is performed by calculating the total useful floor area of residential buildings which are in use. This depends on the demolition and vacancy rate, and can thus be influenced by various scenario parameters. The difference between the demand for living area and the building stock capacity corresponds to the newly built area. For non-residential buildings, national population data are also used as a proxy indicator. However, to account for additional drivers influencing non-residential building construction dynamics, a calibration factor derived from historical construction rates is introduced. The number of buildings constructed is then multiplied by the A1-A5 emissions provided by the MMG-SLiCE model.

Demolition activities are calculated using Weibull probability functions^22,23^. Each year, these functions provide a proportion of building archetypes that are demolished, based on their typology and age. All of the building archetypes that have not undergone a medium or deep renovation can be demolished. This would otherwise be considered unlikely within the time frame. The number of demolished buildings is then multiplied by the C1-C4 emissions provided by the MMG-SLiCE model. Renovations are implemented using the renovation rates specified in the scenarios. The number of renovated buildings is then multiplied by the B5 emissions provided by the MMG-SLiCE model. Emissions from maintenance, replacement and operational energy use are averaged across the reference study period of the building. At the stock level, the annual maintenance, replacement and energy use are calculated by the model, assuming that, for every year, these operations are needed for occupied or secondary buildings (empty dwellings are not considered for these annual emissions). Both the building stock activities and emission data are influenced by scenarios including GHG emission reduction strategies.

### Calibration and validation of the model

The building stock model and its data are both calibrated and validated to ensure representativeness. Calibration is performed for new building construction, thereby directly affecting upfront embodied emissions, and for operational emissions. Together, they represent a large majority of the life cycle emissions of the building stock in the baseline year (2020). In particular, new building constructions are calibrated for the baseline year to reach 80% of the delivered building permits^65^, averaged between 2014 and 2023 as a reference value. This is meant to consider a 20 % difference between the number of building permits and the actual number of buildings built (as not all permits are actually built). Regarding operational emissions (B6) a calibration for the baseline year (2020) is also performed, using reference emissions for 2020 from the BSO^21^. Direct emissions are overtaken as provided and, due to differences in modeling scope, only indirect emissions for HVAC are considered (other electricity uses are not included).

Validation of the model is performed in two steps. First, the archetype-related attributes underwent multiple validation rounds by regional experts who were part of the EU-WLC project^48^. Additionally, an existing, peer-reviewed model is used to critically review and verify the validity of the modeling results obtained from the main modeling pipeline, for the baseline year and the BAU scenario (see Supplementary Note 2). The established MESSAGEix-Buildings Model for Energy Supply Strategy Alternatives and their General Environmental Impact, a bottom-up building sector model to assess energy, material demands, and GHG emissions of buildings at the regional and global scales under different socioeconomic, technological, climate and policy scenarios, as well as the STURM Building Stock Turnover Model, a model based on dynamic material flow analysis to assess new constructions, demolitions and renovation activities, are deployed for generating those additional validation runs^22,23^, as shown in Supplementary Figs. 7 and 8. The comparison demonstrates that the PULSE-EU produces results within a reasonable and credible range when benchmarked against an independently developed, peer-reviewed framework. Minor discrepancies can be attributed to differences in data sources, modeling assumptions, and structural formulations between the two models.

### Scenario modeling of greenhouse gas emission reduction strategies

The modeling of future scenarios in this study is based on the combination of different settings for the implementation of GHG emission reduction strategies, meaning that it relies on the implementation of specific strategies to reduce the life cycle GHG emissions of EU buildings. These strategies have been identified based on literature review and stakeholder consultation^14^, and further grouped into six main categories: (1) the implementation of circularity measures (including the extension of the service life of buildings through renovation, vacancy reduction, efficient structural material use, as well as increased reuse and recycling facilitated by on-site waste sorting), (2) the reduction of per capita space demand (by increasing the use intensity of residential buildings), (3) the shift to low carbon, bio-based materials (through timber construction and bio-based insulation), (4) the reduction of operational emissions (including thermal renovation, exchange in HVAC systems, increased share of renewable energy in the electricity and district heating, as well as the reduction in temperature setpoints), (5) the improvement of material production processes (through efficiency improvements, the use of alternative fuels and innovative technologies in material production, such as carbon capture and storage) and (6) the improvement of transport and construction processes (including alternative fuels use, machine optimization and reduction in transport distances).

The scenarios are modeled through different settings of key strategy parameters representing their diffusion level, meaning different ambition levels for the uptake of individual strategies across MS (see Supplementary Note 1). In particular, the potential diffusion of each strategy is quantified for three key decades (2030, 2040, 2050) according to three levels of ambition (low, medium, and high). For example, the reduction of per capita space demand is estimated to be 20 % lower in 2050 compared to 2020 with a high ambition level^66^. The capacity of MS to implement these strategies is also qualitatively assessed according to the low, medium and high approach. For instance, while Austria might have a high capacity to implement a specific strategy in 2040, Portugal might only have a medium capacity, and vice-versa. This allows for the consideration of local conditions in the scenario modeling. They were defined by analyzing suitability condition criteria reported in Supplementary Table 5 (e.g., roundwood production in the case of bio-based materials) and discussing with stakeholders^14^. The different strategies are then activated up to these capacity levels (or beyond) to simulate various scenarios, in particular the ones reaching the EU policy targets.

The EU policy targets used for the scenario narratives were gathered by reviewing the main EU policy documents. Country-specific policies and GHG emission reduction targets were not specifically included, which is a limitation of this approach. Due to their cross-sectoral nature, building-related emissions are impacted by various EU policies, even if they do not directly target them. A comprehensive overview of the EU policy framework^67^ shows that buildings are impacted by existing targets from emissions policies, such as the ETS, energy policies, such as the EED or the RED, direct building and construction policies, especially the EPBD, and end-of-life policies, such as the Waste Framework Directive. Most of the policy targets are formulated for the year 2030. Compared to 2020, they include, for example, an improvement of the energy efficiency of the building stock of 16 %, associated with a decrease of 31 % in operational GHG emissions, and a decrease of 45 % in the GHG emissions from the production of ETS-regulated construction materials, such as steel or concrete. These were all considered for the scenarios (see Supplementary Table 7), however, apart from the EPBD, they are formulated for the whole economy, not specifically for buildings.

When generating the scenarios, especially the HOPE scenario, the relevant strategies to reach these policy targets were gradually activated until the targets were reached. The total GHG emissions or energy consumption of the EU were compared to these EU policy targets, but not the ones from the MS. This allows, for example, for MS with higher capacity to reduce emissions faster than others, while still ensuring the overall EU target is achieved.

### Reporting summary

Further information on research design is available in the Nature Portfolio Reporting Summary linked to this article.

## Supplementary information

Supplementary Information

Reporting Summary

Transparent Peer Review file

## Data Availability

The minimum dataset required to interpret, verify and extend the results of this study comprises (i) the archetype-related datasets (life cycle scenario definitions, inventories and impact assessment results), (ii) the scenario input data (the detailed list of greenhouse gas mitigation strategies, the input data used for their implementation and the Member State implementation capacities), and (iii) the scenario output data. Archetype-related data generated in this study have been deposited in the KU Leuven Research Data Repository^59,60,68^. These files are openly available under the CC BY 4.0 license. Scenario input data are available in a separate publication^14^ under CC BY-NC-ND 4.0 license, and as an archival snapshot of the PULSE‑EU model inputs^16^. Scenario output data generated in this study are can be interactively explored and downloaded via the WLC‑Scenario‑Explorer^24^ under the CC BY 4.0 license. Source data underlying the figures are provided in the Supplementary Information file of this article. Full life cycle impact assessment (LCIA) results at detailed resolution contain third‑party content from the ecoinvent database and are therefore available under restricted access to comply with the ecoinvent End‑User License Agreement. Access is restricted to researchers who hold a current ecoinvent license for version v3.6, cut‑off and can be granted solely for the purpose of reproducing the findings of this study. To request access, contact the corresponding authors with (i) proof of a valid ecoinvent license, (ii) institutional affiliation, and (iii) a brief description of the intended use; approved requesters will be asked to agree to a non‑redistribution and non‑commercial use condition in line with the ecoinvent license. Upon approval, access will be provided via a secure link.

## Code availability

The PULSE-EU model^16^, which is used to upscale the building archetypes to the stock level and projects the future scenarios, is open-source at https://github.com/ITE-NHB/PULSE-EU under the Graz University of Technology Software License Agreement, and is archived at 10.5281/zenodo.19550009.
