# Supplementary material for: Context-specific life cycle emissions pathways for EU buildings and construction
Source: Nat Commun. 2026 May 25;17:6804. doi: 10.1038/s41467-026-73433-1 (PMC13385351; doi:10.1038/s41467-026-73433-1)
Supplement: Supplementary file 1 — Supplementary Information [file 41467_2026_73433_MOESM1_ESM.pdf]

Supplementary Information to

Context-specific life cycle emissions pathways for EU buildings and construction

Alaux Nicolas[1], Bechstedt Nicolas[1], Zhong Xiaoyang[2,3], Mastrucci Alessio[2], Ramon Delphine[4], Steinberger-Maierhofer Dominik[1], Allacker Karen[4*], Passer Alexander[1*], Röck Martin[4,5]

[1] Graz University of Technology, Institute of Structural Design, Working Group Sustainable Construction, Graz, Austria.

[2] Energy, Climate, and Environment (ECE) Program, International Institute for Applied Systems Analysis (IIASA), Laxenburg, Austria.

[3] Tsinghua University, Tsinghua Shenzhen International Graduate School, Institute of Environment and Ecology, Shenzhen, China.

[4] KU Leuven, Faculty of Engineering Science, Department of Architecture, Leuven, Belgium.

[5] RISE Institute for Regenerative Spatial Systems Science, Vienna, Austria.

[*] Corresponding authors: Karen Allacker (karen.allacker@kuleuven.be) and Alexander Passer (alexander.passer@tugraz.at)

List of Supplementary Items

- **Supplementary Notes**
  - **Supplementary Note 1:** Page 3
  - **Supplementary Note 2:** Page 4
- **Supplementary Figures**
  - **Supplementary Figure 1:** Page 4
  - **Supplementary Figure 2:** Page 5
  - **Supplementary Figure 3:** Page 5
  - **Supplementary Figure 4:** Page 6
  - **Supplementary Figure 5:** Page 6
  - **Supplementary Figure 6:** Page 7
  - **Supplementary Figure 7:** Page 7
  - **Supplementary Figure 8:** Page 8
- **Supplementary Tables**
  - **Supplementary Table 1:** Page 8
  - **Supplementary Table 2:** Page 8
  - **Supplementary Table 3:** Pages 8,9
  - **Supplementary Table 4:** Page 10
  - **Supplementary Table 5:** Pages 10,11
  - **Supplementary Table 6:** Page 11
  - **Supplementary Table 7:** Page 11
  - **Supplementary Table 8:** Pages 11,12,13
  - **Supplementary Table 9:** Pages 13,14
- **Supplementary References:** Page 14

Supplementary Note 1: Additional information regarding the modelling of future scenarios

The modelling of future scenarios is measure-driven, meaning that it relies on the implementation of specific strategies to reduce the whole life greenhouse (GHG) emissions of buildings in the European Union (EU).

These strategies have been identified based on literature review and stakeholder consultation[1], and further grouped into six main categories or packages: (1) the implementation of circularity measures, (2) the reduction of per capita space demand, (3) the shift to low carbon, bio-based materials, (4) the reduction of operational emissions, (5) the improvement of material production processes and (6) the improvement of transport and construction processes. The link between the strategies from the original article[1] and this contribution is shown in Supplementary Table 4.

For each of these strategies, the capacity of each Member State (MS) to implement them was also qualitatively assessed, based on a list of criteria and indicators (provided in Supplementary Table 5), allowing for consideration of local conditions within the scenario modelling. This results in a low, medium or high capacity for each MS to implement each strategy, in each decade (2020/2030/2040/2050), as shown in Supplementary Figure 6.

Based on this framework, the scenarios are generated using four activation levels for each of the six strategies: (1) strategy not activated, (2) strategy activated to half the capacity of the MS, (3) strategy activated to the full capacity of the MS and (4) strategy activated to full capacity assuming all MS have a high capacity (e.g. beyond the identified capacity). These scenarios can be activated individually using the online whole life carbon scenario explorer[2], leading to 4,096 possibilities. The quantitative scenario emissions results used for Figure 1 in the main text can be retrieved this online tool[2]. The equivalence between the scenario names used in the paper and the ones from the tool is in Supplementary Table 6.

Due to their cross-sectoral nature, building-related emissions are impacted by various EU policies, even if they do not directly target them, as shown in Supplementary Table 7. These policies are the ones which were considered for the scenario narratives. A comprehensive overview of the EU policy framework[3] shows that buildings are impacted by existing targets from emissions policies, such as the Emissions Trading System (ETS), energy policies, such as the Energy Efficiency Directive (EED) or the Renewable Energy Directive (RED), direct building and construction policies, especially the Energy Performance of Buildings Directive (EPBD), and end-of-life policies, such as the Waste Framework Directive (WFD). Most of the policy targets are formulated for the year 2030 and compared to 2020 (details available in Supplementary Table 7). These are ambitious, however, apart from the EPBD, they are formulated for the whole economy, not specifically for buildings.

When generating the scenarios, the relevant strategies to reach these policy targets were gradually activated until the targets were reached. The implementation of each strategy in the policy scenarios is as follows (note that the number of these strategies refer to the ones from Alaux et al. (2024)[1] as shown in Supplementary Figure 6 and Supplementary Table 4):

- **BAU (Business-As-Usual)**: No strategy activated (targets not reached).
- **HOPE (Honoring Official Policy Expectations)**: Strategies 5 and 9 activated to the maximum level (high for all MS), strategies 6, 7, 8 and 10 activated to half the MS capacity. All targets reached.
- **COPE (Capacity-Orchestrated Policy Execution)**: Strategies 5 and 9 activated to the maximum capacity of each MS (low, medium or high), strategies 6, 7, 8 and 10 activated to half the capacity of each MS. Targets are partially reached (only EPBD, RED and WFD).
- **SMART (Strategy Mix Approach for Robust Trajectories)**: All strategies are activated to the maximum capacity of each MS (low, medium or high). Targets are implicitly reached (e.g., the emissions in 2030 for which the targets are specified are lower than in the HOPE scenario).

The quantitative data used for the modelling of these strategies, including diffusion rates and GHG emission reduction potentials, are available on GitHub publication of the model.

Supplementary Note 2: Validation with the MESSAGEix-Buildings model

For validation of the results generated with the PULSE-EU (Prospective Upscaling of Life cycle Scenarios and Environmental impacts for EU buildings) model, we ran the BAU scenario for residential buildings using the peer-reviewed MESSAGEix-Buildings model[4,5]. To enable a direct comparison, we calibrated the base year for both models, allowing a consistent basis for evaluating future projections.

As shown in Supplementary Figure 7, the projected total residential floor area in 2050 under the BAU scenario is broadly aligned across all regions between the two models, demonstrating comparable scale and growth patterns. Supplementary Figure 8 further compares the associated embodied and operational emissions, which also show consistency in terms of regional distribution and directional trends.

The comparison demonstrates that the model produces results within a reasonable and credible range when benchmarked against an independently developed, peer-reviewed framework. Minor discrepancies can be attributed to differences in data sources, model assumptions, and structural formulations between the two models. Where applicable, the MESSAGEix-Buildings runs adopt the same input data as the PULSE-EU model, particularly for current building stocks, building lifetimes, and population projections.

Additional data inputs and full modelling details of the MESSAGEix-Buildings model are available in previous publications[4,5] and on GitHub.

Supplementary Figures

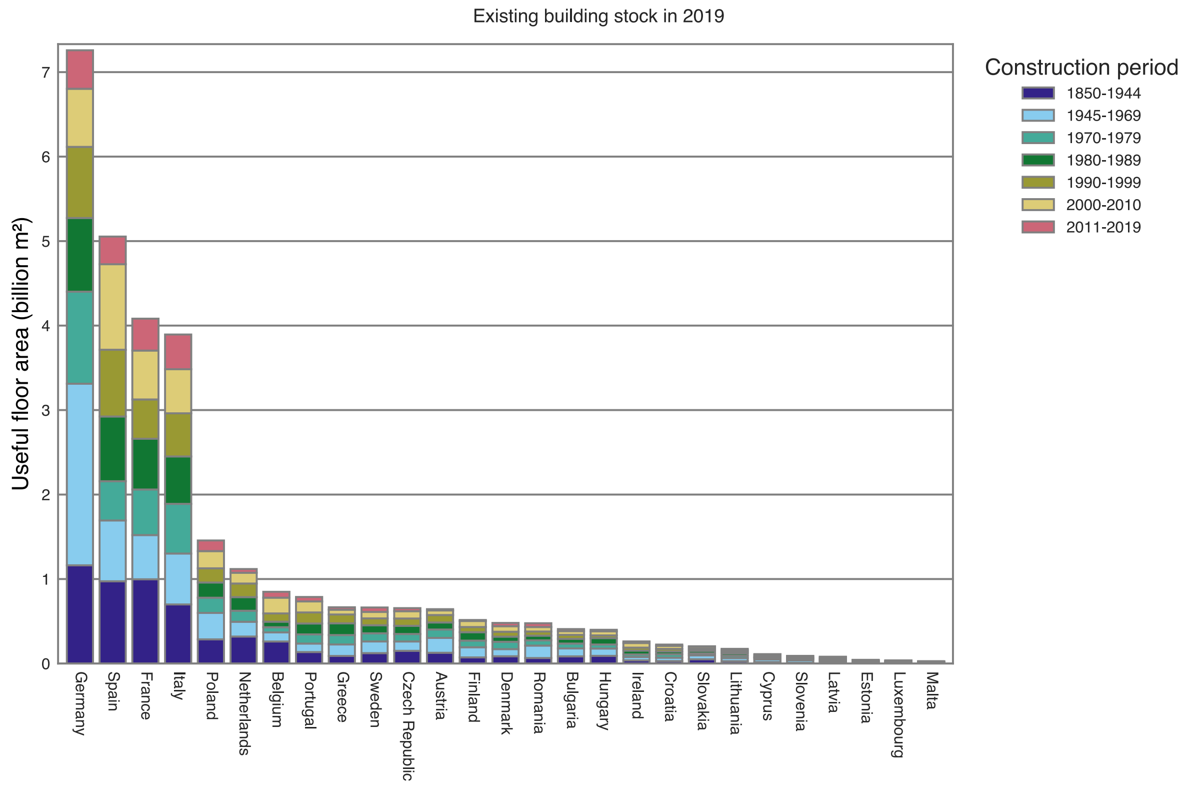

Supplementary Figure 1. Building stock characterization and aggregation based on archetypes: Total useful area of the existing building stock in 2019 per construction period and per country. This figure was generated with python using matplotlib (v3.10.8) and polars (v1.39.3).

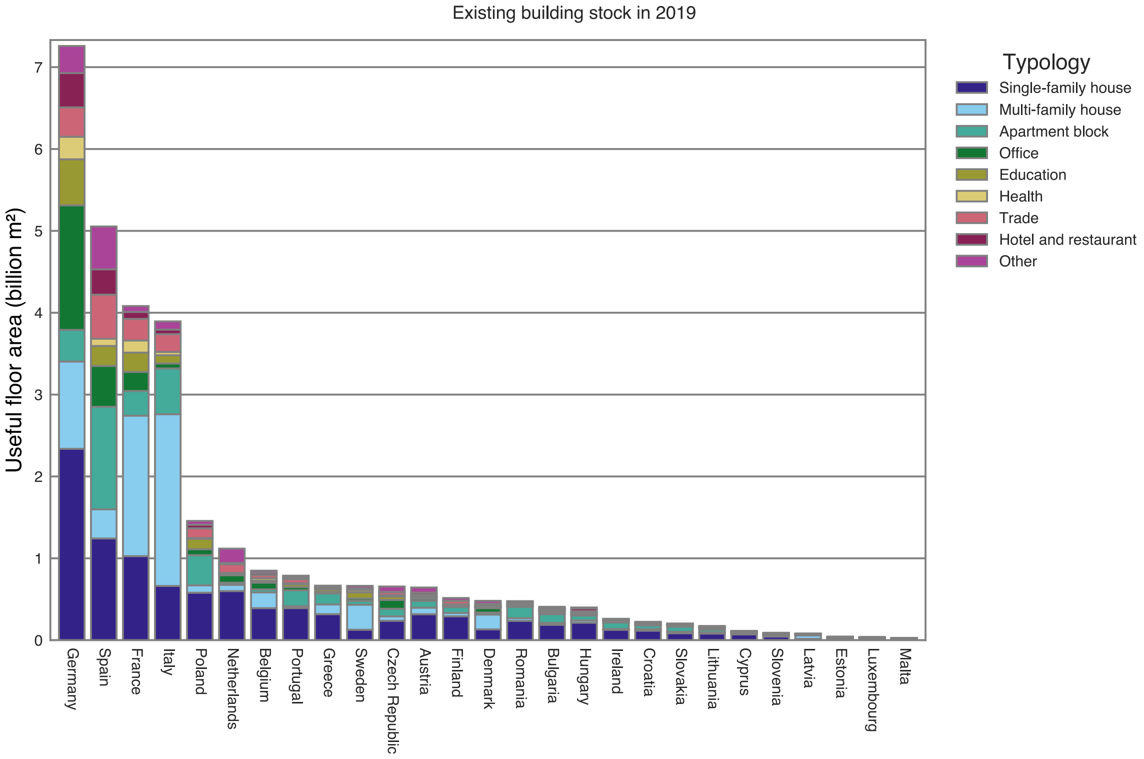

Supplementary Figure 2. Building stock characterization and aggregation based on archetypes: Total useful area of the existing building stock in 2019 per typology and per country. This figure was generated with python using matplotlib (v3.10.8) and polars (v1.39.3).

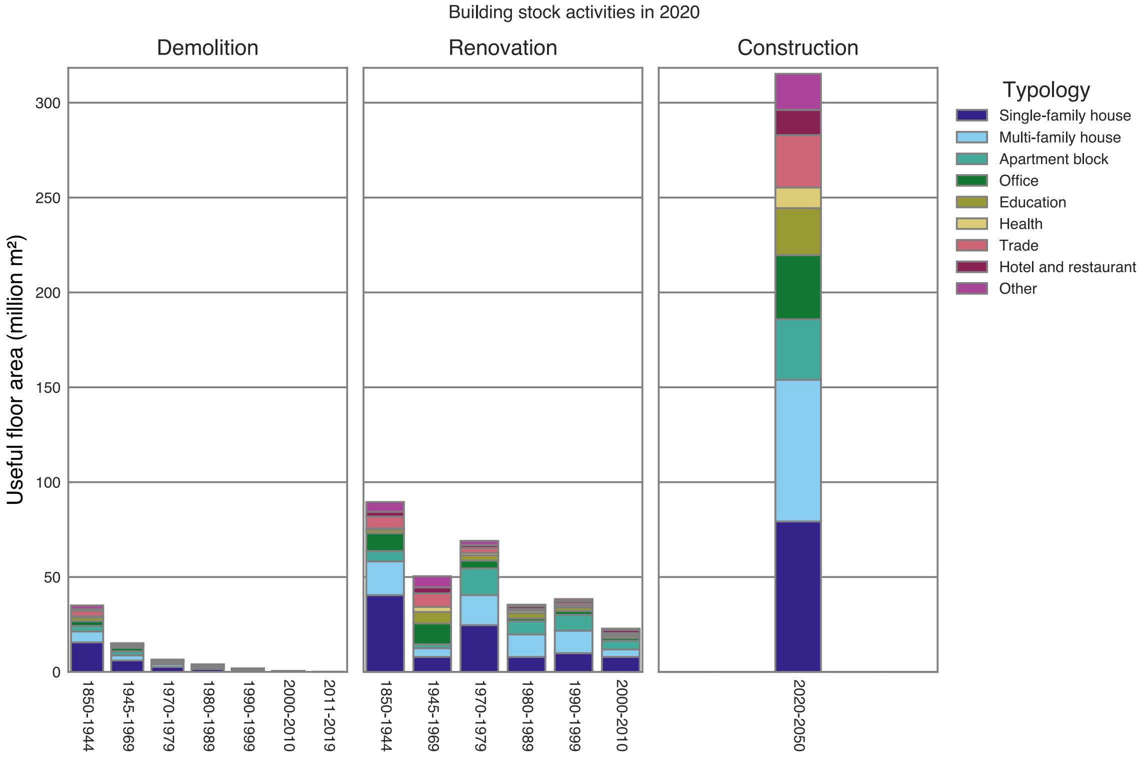

Supplementary Figure 3. Baseline year (2020) building stock activity results (construction, renovation, demolition) in the European Union, in useful floor area and per building typology, using the PULSE (Prospective Upscaling of Life cycle Scenarios and Environmental impacts) modelling logic[6]. This figure was generated with python using matplotlib (v3.10.8) and polars (v1.39.3).

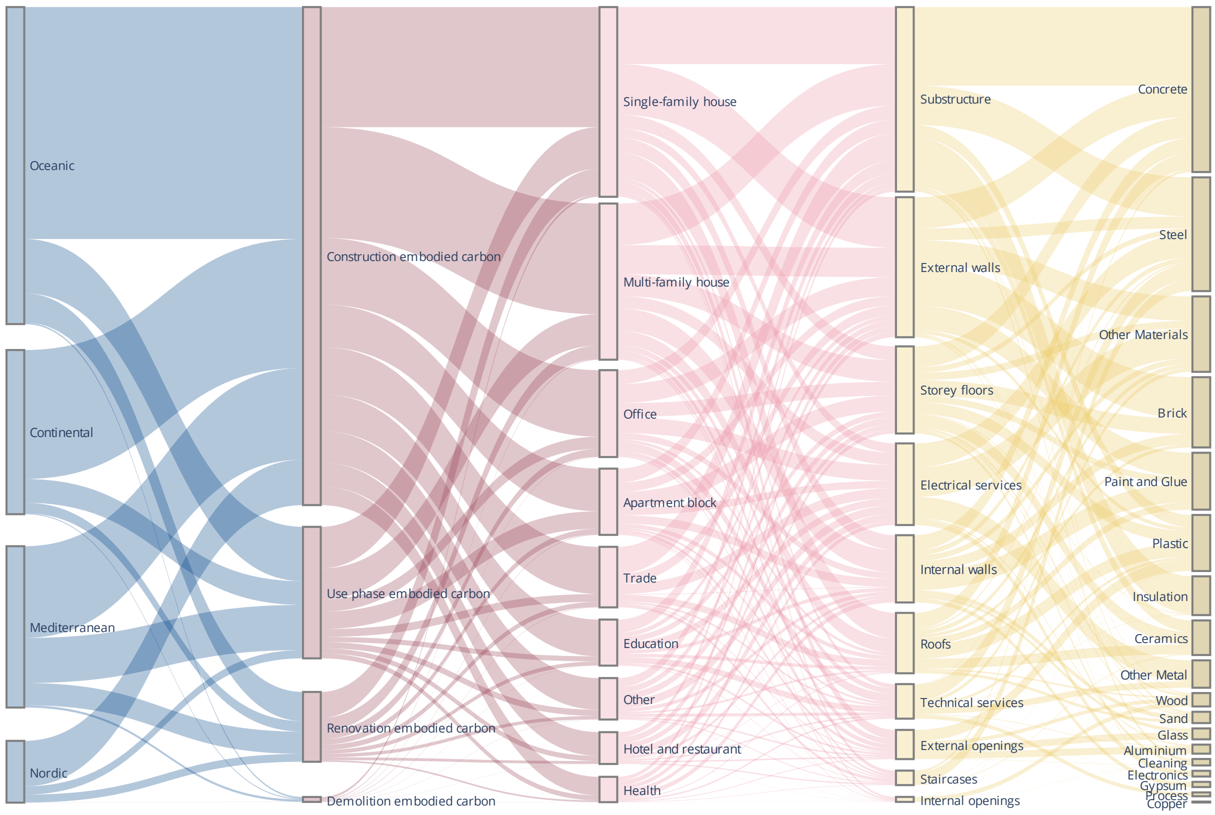

Supplementary Figure 4. Origin of embodied greenhouse gas (GHG) emissions in the baseline year (2020) across countries, life cycle stage, building typologies, building components and materials. These are calculated using fossil GHG emissions only, to avoid potential issues in representing negative emission flows. This figure was generated with python using plotly (v6.3.0), python-kaleido (v1.2.0) and polars (v1.39.3).

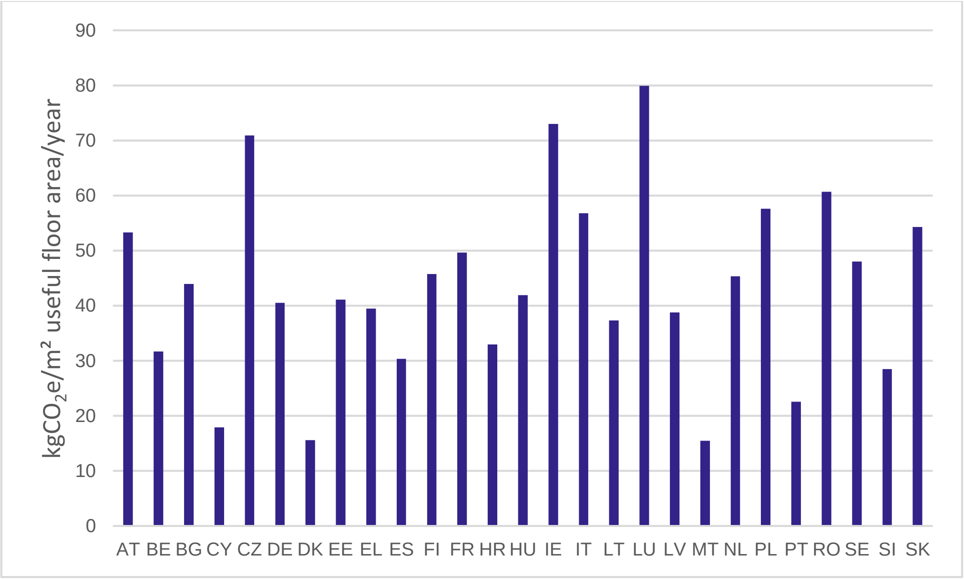

Supplementary Figure 5. Operational greenhouse gas (GHG) emissions of the apartment buildings archetypes, averaged across the different construction periods (weighted based on their useful floor area). This figure was generated with Microsoft Excel 2019.

Country	1. Reduce the per capita space demand				2. Prioritise better use, renovation and repair over demolition and new construction				3. Optimize the use of materials				4. Increase use of bio-based materials				5. Reduce emissions from traditionally high-impact construction materials			
	Baseline	2030	2040	2050	Baseline	2030	2040	2050	Baseline	2030	2040	2050	Baseline	2030	2040	2050	Baseline	2030	2040	2050
Austria	M	M	M	M	M	M	H	H	M	H	H	H	M	H	H	H	M	M	M	M
Belgium	M	M	M	M	M	M	M	H	M	M	H	H	M	M	H	H	M	M	M	M
Bulgaria	L	L	L	L	M	M	M	M	M	M	M	H	M	M	M	M	M	M	M	M
Croatia	L	L	L	L	M	M	M	M	L	L	M	M	L	L	M	M	M	M	M	M
Cyprus	M	M	M	M	L	L	L	L	L	L	M	M	L	L	L	L	L	L	M	M
CzecHRepublic	L	L	L	L	M	M	M	M	M	M	M	H	H	H	H	H	M	M	M	M
Denmark	H	H	H	H	H	H	H	H	H	H	H	H	M	M	H	H	H	H	H	H
Estonia	L	L	L	L	L	M	M	M	M	M	M	H	H	H	H	H	M	M	M	M
Finland	M	M	M	M	M	M	M	H	M	H	H	H	H	H	H	H	M	M	M	H
France	M	M	M	M	H	H	H	H	H	H	H	H	M	H	H	H	M	M	H	H
Germany	M	M	M	M	M	H	H	H	H	H	H	H	M	H	H	H	M	M	M	M
Greece	L	L	L	L	L	L	M	M	L	L	M	M	L	L	L	L	L	L	M	M
Hungary	L	L	L	L	L	M	M	M	M	M	M	M	M	M	M	H	L	L	L	L
Ireland	L	L	L	L	M	M	H	H	M	M	H	H	M	M	H	H	L	M	M	M
Italy	M	M	M	M	M	M	M	H	L	M	M	H	M	M	H	H	M	M	M	M
Latvia	L	L	L	L	L	L	M	M	L	L	M	M	M	M	H	H	L	L	L	L
Lithuania	M	M	M	M	L	L	M	M	L	L	M	M	M	M	H	H	L	L	L	L
Luxembourg	H	H	H	H	M	M	M	H	H	H	H	H	M	M	H	H	M	M	M	M
Malta	L	L	M	M	L	L	M	M	L	L	M	M	L	L	L	L	L	L	L	L
Netherlands	M	M	H	H	H	H	H	H	H	H	H	H	M	M	H	H	M	H	H	H
Poland	L	L	M	M	M	M	M	H	M	M	M	H	M	H	H	H	M	M	M	H
Portugal	M	M	M	M	L	M	M	M	L	M	M	H	M	M	M	M	L	M	M	M
Romania	L	L	L	L	M	M	M	M	L	L	L	M	M	M	H	H	M	M	M	M
Slovakia	L	L	L	M	M	M	M	H	M	M	M	H	M	M	H	H	M	M	M	H
Slovenia	M	M	M	M	M	M	M	M	M	M	M	H	M	H	H	H	M	M	M	H
Spain	M	M	M	M	M	M	M	M	M	M	M	H	M	M	M	M	M	M	M	H
Sweden	H	H	H	H	H	H	H	H	H	H	H	H	H	H	H	H	M	H	H	H

Country	6. Reduce emissions from the transport of construction materials				7. Reduce emissions at construction site				8. Increase circular material use				9. Reduce operational greenhouse gas emissions				10. Reduce construction and demolition waste			
	Baseline	2030	2040	2050	Baseline	2030	2040	2050	Baseline	2030	2040	2050	Baseline	2030	2040	2050	Baseline	2030	2040	2050
Austria	H	H	H	H	M	H	H	H	M	H	H	H	M	M	M	H	M	H	H	H
Belgium	M	H	H	H	M	H	H	H	M	H	H	H	M	H	H	H	M	H	H	H
Bulgaria	M	M	M	H	M	M	M	H	L	M	M	H	M	M	M	H	L	M	M	H
Croatia	M	M	H	H	M	M	H	H	L	L	M	H	M	M	M	H	L	L	M	H
Cyprus	M	M	M	M	L	L	M	M	L	L	L	M	L	L	M	M	L	L	M	M
CzecHRepublic	M	M	H	H	M	M	H	H	M	M	H	H	M	M	H	H	M	M	M	H
Denmark	M	H	H	H	H	H	H	H	H	H	H	H	M	M	M	M	H	H	H	H
Estonia	M	M	H	H	M	M	M	H	M	H	H	H	M	M	M	H	M	M	H	H
Finland	M	M	H	H	M	M	M	H	M	H	H	H	M	M	M	H	M	M	H	H
France	M	M	H	H	H	H	H	H	M	H	H	H	M	M	H	H	M	M	H	H
Germany	M	H	H	H	H	H	H	H	H	H	H	H	M	M	H	H	H	H	H	H
Greece	M	M	M	M	L	L	M	M	L	L	M	H	M	M	M	H	L	L	M	M
Hungary	L	L	M	H	M	M	M	H	L	M	M	H	M	M	M	M	L	M	M	H
Ireland	M	H	H	H	H	H	H	H	M	M	H	H	L	L	M	M	M	H	H	H
Italy	M	M	H	H	M	M	H	H	M	M	H	H	M	M	H	H	M	M	H	H
Latvia	L	M	M	H	L	L	M	M	L	L	M	H	M	M	M	H	L	L	M	H
Lithuania	L	M	M	H	L	L	M	M	L	M	M	H	M	M	M	H	M	M	M	H
Luxembourg	M	M	M	H	H	H	H	H	H	H	H	H	M	M	M	H	H	H	H	H
Malta	L	L	L	M	L	L	M	M	L	L	M	M	L	M	M	M	L	L	M	M
Netherlands	M	M	H	H	H	H	H	H	H	H	H	H	M	M	H	H	H	H	H	H
Poland	H	H	H	H	M	M	M	H	L	M	M	H	M	M	H	H	L	M	M	H
Portugal	H	H	H	H	L	M	M	H	M	M	H	H	M	M	H	H	M	M	M	H
Romania	M	M	M	H	M	M	M	H	L	L	M	H	M	M	M	H	L	L	M	H
Slovakia	M	M	H	H	M	M	M	H	L	M	M	H	M	M	M	H	L	M	M	H
Slovenia	M	M	M	H	M	M	M	H	M	M	H	H	M	M	M	M	M	M	H	H
Spain	H	H	H	H	M	M	M	H	M	H	H	H	M	M	H	H	M	H	H	H
Sweden	M	M	H	H	H	H	H	H	M	H	H	H	M	H	H	H	M	H	H	H

Supplementary Figure 6. Capacity of the Member States to implement each strategy (L = low capacity, M = medium capacity, H = high capacity), adapted from Alaux et al. (2024)[1]. This figure was generated with Microsoft Excel 2019.

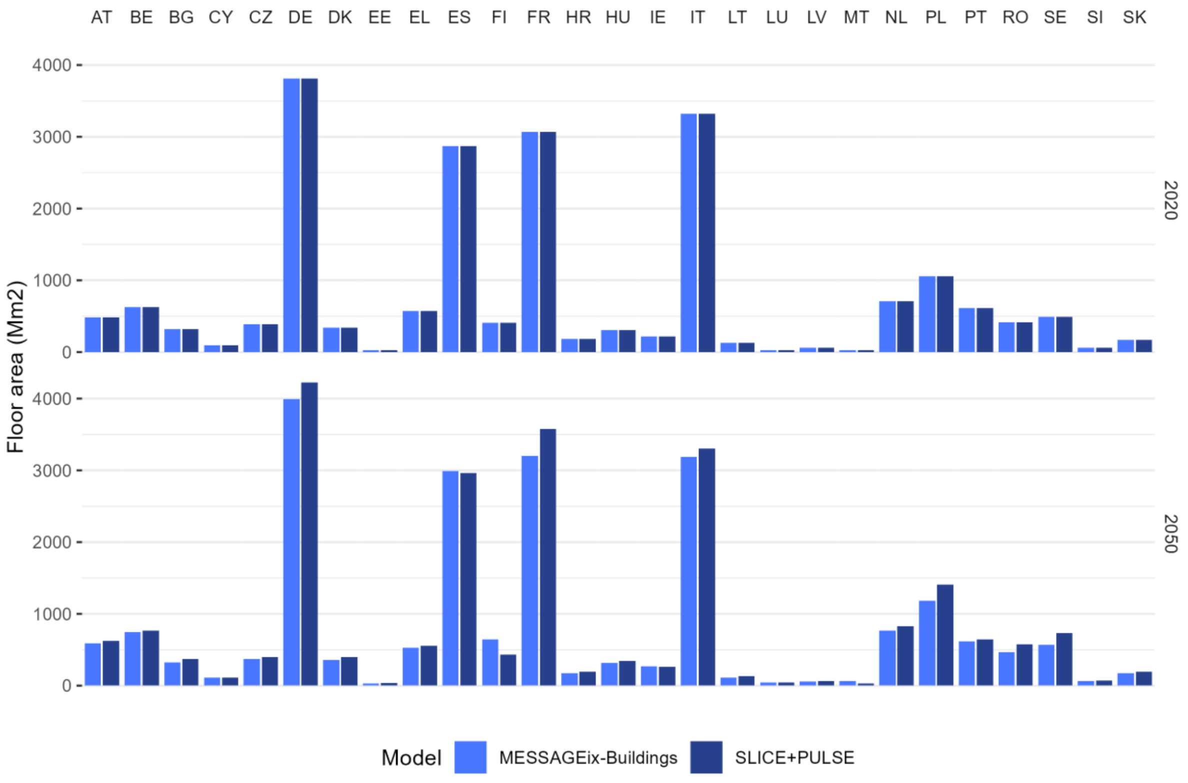

Supplementary Figure 7. Residential building useful floor area in 2020 and 2050 under the Business-As-Usual scenario, generated with the MESSAGEix-Buildings model[4,5] and compared with the PULSE-EU model (named SLICE+PULSE on the graph). This figure was generated with python using matplotlib (v3.10.8).

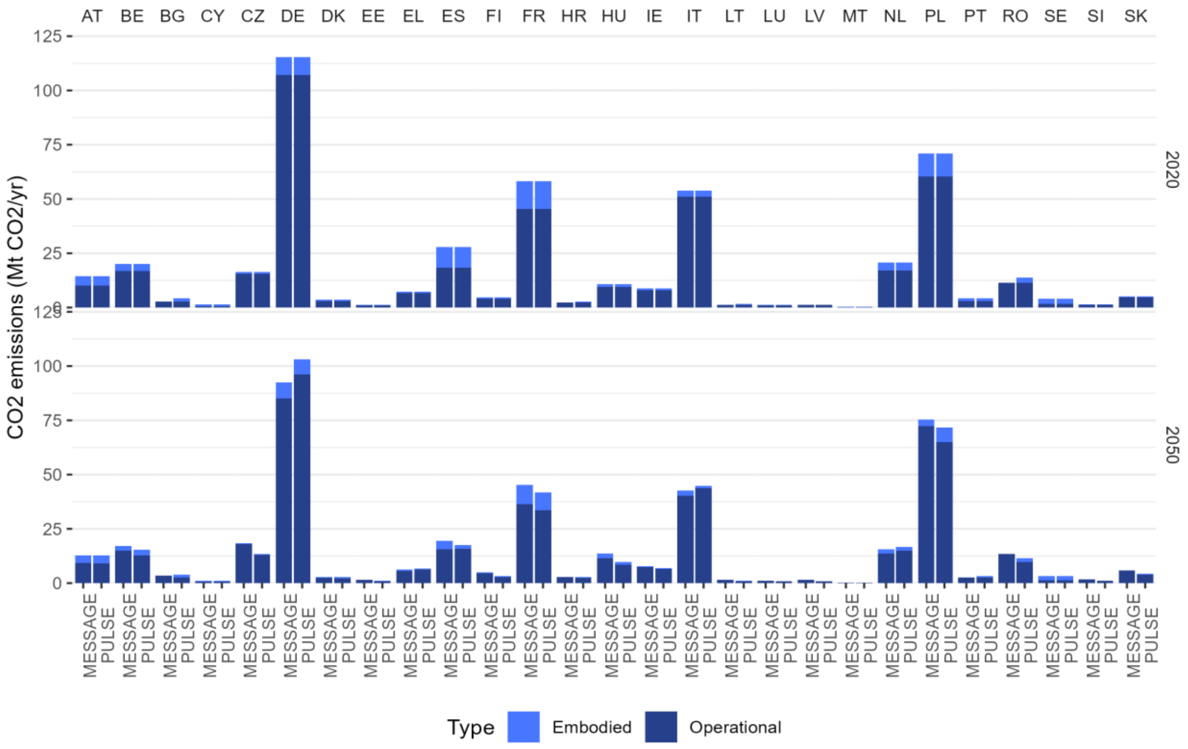

Supplementary Figure 8. Embodied and operational emissions for residential buildings in 2020 and 2050 under the Business-As-Usual scenario, generated with the MESSAGEix-Buildings model[4,5] and compared with the PULSE-EU model (named PULSE on the graph) This figure was generated with python using matplotlib (v3.10.8)

Supplementary Tables

Supplementary Table 1. Number of archetypes created for the model. A total of 15,026 archetypes were created for the European Union (EU) building stock, categorized into existing buildings archetypes, built until 2019 (4,490), new buildings archetypes, built as of 2020 (5,919) and thermal renovation archetypes for existing buildings (4,616).

Archetype category	Number of archetypes for the EU
Existing buildings	4,490
New buildings	5,919
Building renovation	4,617

Supplementary Table 2. Classification of European Union Member States into climatic zones.

Zone	Country	Zone	Country	Zone	Country	Zone	Country
Oceanic	Belgium	Continental	Austria	Mediterranean	Croatia	Nordic	Denmark
Oceanic	France	Continental	Bulgaria	Mediterranean	Cyprus	Nordic	Estonia
Oceanic	Germany	Continental	Czech Republic	Mediterranean	Greece	Nordic	Finland
Oceanic	Ireland	Continental	Hungary	Mediterranean	Italy	Nordic	Latvia
Oceanic	Luxembourg	Continental	Poland	Mediterranean	Malta	Nordic	Lithuania
Oceanic	Netherlands	Continental	Romania	Mediterranean	Portugal	Nordic	Sweden
		Continental	Slovakia	Mediterranean	Spain		
		Continental	Slovenia				

Supplementary Table 3. Underlying data for Figure 2 in main text. This table presents per capita embodied and operational greenhouse gas (GHG) emissions for Member States (MS) of the European

Union (EU) in 2020, and the projected per capita life cycle GHG emission reductions by 2030 and 2050 under the SMART (Strategy Mix Approach for Robust Trajectories) scenario compared to BAU (Business-As-Usual), highlighting potential shared reduction efforts. It is divided into: a) Embodied GHG emissions per capita for each individual MS in 2020 (in tons of carbon dioxide equivalent per capita, or tCO_2e/cap). b) Operational GHG emissions per capita for each individual MS in 2020 (in tCO_2e/cap). c) Reduction in life cycle GHG emissions between SMART and BAU for 2030, per capita (in tCO_2e/cap). d) Reduction in life cycle GHG emissions between SMART and BAU for 2050, per capita (in tCO_2e/cap). c) and d) were obtained by subtracting the GHG emissions of the BAU scenario from the ones of the SMART scenario (in 2030 or 2050), and then dividing by the population in the respective year. This indicates how the GHG emission reduction effort can be shared among EU MS in the SMART scenario. In all graphs, the value in the circle where EU is written represents the average value across all EU MS. Country abbreviations use EU codes per the Interinstitutional Style Guide.

	**a) Embodied 2020 (in tCO_2e/cap)**	**b) Operational 2020 (in tCO_2e/cap)**	**c) Reduction 2030 (in tCO_2e/cap)**	**d) Reduction 2050 (in tCO_2e/cap)**
AT	0.92	1.5	0.46	1.19
BE	0.76	2.07	0.54	2.03
BG	0.52	0.58	0.38	0.76
CY	1.46	1.24	0.19	0.76
CZ	0.35	2.18	0.74	2.00
DE	0.41	1.84	0.44	1.30
DK	0.62	0.78	0.43	0.76
EE	0.71	1.52	0.68	2.28
EL	0.23	0.8	0.10	0.57
ES	0.55	0.62	0.15	0.47
FI	0.73	1.29	0.23	0.95
FR	0.61	1.03	0.39	1.05
HR	0.35	0.78	0.22	0.90
HU	0.39	1.32	0.22	1.07
IE	0.47	2.11	0.27	1.19
IT	0.23	1.3	0.23	0.91
LT	0.69	0.65	0.15	0.72
LU	0.99	2.73	0.83	2.25
LV	0.41	1.08	0.27	1.28
MT	0.77	0.69	0.28	0.97
NL	0.59	1.48	0.49	1.39
PL	0.60	2.28	0.57	2.90
PT	0.43	0.44	0.13	0.42
RO	0.29	0.76	0.17	0.89
SE	0.58	0.29	0.25	0.48
SI	0.40	0.93	0.21	0.69
SK	0.29	1.25	0.48	1.28
EU	0.48	1.33	0.35	1.17

Supplementary Table 4. Equivalence table between the strategies used in the paper and the strategy numbers from Alaux et al. (2024)[1].

Strategies used in this paper	Description of included measures	Strategy number[1]
Implementation of circularity measures	Extension of the service life of buildings through renovation, vacancy reduction, efficient structural material use, as well as increased reuse and recycling facilitated by on-site waste sorting.	2, 3, 8, 10
Reduction of per capita space demand	Reduce space demand per capita through sufficiency measures by increasing the use intensity of residential buildings.	1
Shift to low carbon, bio-based materials	Increase the shift to bio-based construction materials through timber construction and bio-based insulation.	4
Reduction of operational emissions	Thermal renovation, exchange in Heating, Ventilation and Air Conditioning systems, increased share of renewable energy in the electricity and district heating, as well as the reduction in temperature setpoints.	9
Improvement of material production processes	Efficiency improvements, use of alternative fuels and innovative technologies in material production, such as carbon capture and storage.	5
Improvement of transport and construction process	Alternative fuels use, machine optimization and reduction in transport distances.	6, 7

Supplementary Table 5. List of criteria and indicators used for assessing the suitability conditions of each Member State for each strategy, from Alaux et al. (2024)[1].

Suitability condition criteria	Indicators
Availability of bio-based construction materials	Roundwood production
	Available forest wood stock for the supply per country area
	Number of bio-based industry installations
	Production of cereals and other fast-growing crops
Material recycling	Generated construction waste
	Treatment of construction waste
	Market maturity for construction and demolition waste recovery and recycling
Production capacity for key construction materials	Cement production per capita
	Cement production locations
	Steel production per capita
	Steel production locations
Seismic stability	Average annual economic loss
	Average annual loss of life
Knowledge and skills for low-carbon building design	Initiatives for improving energy efficiency skills in the construction workforce
	Trends in enrolment in tertiary education institutes in the fields of engineering, architecture and construction
Renovation potential	Estimated renovation rates
	Estimated deep renovation rates
	Household renovation spending as a share of the total household disposable income
	Age structure of the building stock
Improvement potential of the buildings stock over the current energy performance	Distribution of Energy Performance Certificate classes
Heating and cooling needs	Average heating degree days
	Average cooling degree days
	Temperature development (1.5°C reference scenario)

Policy framework	Existence of building-related policies beyond the implementation of the Energy Performance of Buildings Directive
	Per capita renovation investments from recovery and resilience plans
Social conditions for low-carbon transition	Gross domestic product per capita
	Household expenditure on housing
	Residential space per capita
	Share of the under-occupied space
	Share of over-crowded dwellings

Supplementary Table 6. Equivalence table between the scenario names used in the paper and the ones used in the online whole life carbon scenario explorer[2].

**Scenario name (paper)**	**Scenario name (scenario explorer)**
BAU (Business-As-Usual)	Predefined: BAU (Business-As-Usual)
HOPE (Honoring Official Policy Expectations)	Predefined: CPOL/A (Optimistic Current Policy Scenario)
COPE (Capacity-Orchestrated Policy Execution)	Predefined: CPOL/B (Conservative Current Policy Scenario)
SMART (Strategy Mix Approach for Robust Trajectories)	Not predefined, but can be manually activated by putting all strategies on the setpoint 1.0.

Supplementary Table 7. Policy targets considered and reached in the HOPE (Honoring Official Policy Expectations) scenario. These targets were compiled from the Energy Performance of Buildings Directive (EPBD), Emissions Trading System (ETS), Renewable Energy Directive (RED), Energy Efficiency Directive (EED) and Waste Framework Directive (WFD).

**Policy**	**Direct building target**	**Considered target (for 2030 compared to 2020)**	**Relevant strategies from Alaux et al. (2024)[1]**
EPBD	Yes	16% improvement in energy efficiency (all buildings).	9
EPBD	Yes	33% improvement in energy efficiency (non-residential).	9
EPBD	Yes	20% improvement in energy efficiency (residential).	9
EPBD	Yes	All new buildings are zero-emission buildings in 2027 (public) and 2030 (all).	9
ETS I	No	45% reduction in embodied emissions (steel, concrete, glass, aluminum, brick).	5, 8
ETS II	No	31% reduction in operational emissions (all buildings).	9
RED	No	42.5% renewable energy mix (all buildings).	9
EED	No	16% reduction in energy consumption (all buildings).	9
WFD	No	70% of non-hazardous waste prepared for reuse or recycling.	6, 7, 8, 10

Supplementary Table 8. Underlying data for Figure 3 in the main text. This table presents the relative change in cumulative greenhouse gas (GHG) emissions when applying a specific strategy, or all of them in the case of the SMART (Strategy Mix Approach for Robust Trajectories) scenario, compared to the BAU (Business-As-Usual) scenario, reported in percent (%). More specifically, the annual GHG emissions over the 2020 to 2050 time period were summed for both the BAU scenario and a scenario in which the strategy is implemented to the capacity of each Member State (MS), and the relative difference between these cumulative emissions was computed. A negative value means a reduction in emissions

for the scenario in which the strategy is implemented, compared to the BAU. Country abbreviations use EU codes per the Interinstitutional Style Guide.

**Country**	**Emission type**	**1**	**2**	**3**	**4**	**5**	**6**	**ALL**
AT	Embodied	-17%	-31%	-8%	36%	-12%	-3%	-22%
AT	Operational	0%	-2%	0%	-29%	0%	0%	-30%
AT	Total	-7%	-13%	-3%	-3%	-5%	-1%	-26%
BE	Embodied	-17%	-22%	-9%	37%	-14%	-3%	-14%
BE	Operational	1%	0%	0%	-48%	0%	0%	-47%
BE	Total	-5%	-7%	-3%	-23%	-4%	-1%	-38%
BG	Embodied	-40%	-17%	-7%	41%	-12%	-2%	-19%
BG	Operational	0%	-1%	0%	-42%	0%	0%	-42%
BG	Total	-19%	-8%	-3%	-2%	-6%	-1%	-31%
CY	Embodied	-12%	-33%	-6%	45%	-5%	-1%	-5%
CY	Operational	1%	-2%	0%	-26%	0%	0%	-26%
CY	Total	-5%	-16%	-3%	8%	-2%	0%	-16%
CZ	Embodied	-19%	-13%	-6%	27%	-10%	-2%	-9%
CZ	Operational	-1%	0%	0%	-50%	0%	0%	-51%
CZ	Total	-3%	-2%	-1%	-40%	-1%	0%	-45%
DE	Embodied	-27%	-20%	-9%	32%	-11%	-3%	-20%
DE	Operational	1%	-2%	0%	-37%	0%	0%	-36%
DE	Total	-5%	-5%	-2%	-23%	-2%	-1%	-33%
DK	Embodied	-26%	-23%	-9%	23%	-23%	-7%	-32%
DK	Operational	-2%	-2%	0%	-36%	0%	0%	-36%
DK	Total	-14%	-12%	-5%	-6%	-12%	-3%	-34%
EE	Embodied	-15%	-7%	-20%	12%	-10%	-10%	-36%
EE	Operational	-2%	-1%	1%	-44%	0%	0%	-44%
EE	Total	-5%	-3%	-5%	-29%	-3%	-3%	-42%
EL	Embodied	-16%	-21%	-3%	73%	-9%	-1%	36%
EL	Operational	-1%	-1%	0%	-30%	0%	0%	-31%
EL	Total	-4%	-5%	-1%	-7%	-2%	0%	-16%
ES	Embodied	-39%	-18%	-3%	36%	-12%	-3%	-12%
ES	Operational	-1%	-1%	0%	-33%	0%	0%	-33%
ES	Total	-17%	-8%	-1%	-3%	-5%	-1%	-24%
EU	Embodied	-24%	-23%	-7%	28%	-15%	-3%	-23%
EU	Operational	0%	-1%	0%	-38%	0%	0%	-37%
EU	Total	-6%	-7%	-2%	-20%	-4%	-1%	-34%
FI	Embodied	-10%	-6%	-8%	36%	-12%	-2%	4%
FI	Operational	0%	0%	0%	-34%	0%	0%	-34%
FI	Total	-4%	-3%	-3%	-6%	-5%	-1%	-19%
FR	Embodied	-26%	-29%	-6%	14%	-25%	-3%	-44%
FR	Operational	-1%	0%	0%	-36%	0%	0%	-36%
FR	Total	-11%	-12%	-2%	-15%	-10%	-1%	-39%
HR	Embodied	-24%	-17%	-7%	27%	-12%	-2%	-19%
HR	Operational	0%	-1%	0%	-27%	0%	0%	-29%
HR	Total	-8%	-6%	-2%	-11%	-4%	-1%	-26%
HU	Embodied	-12%	-13%	-6%	17%	-6%	-2%	-16%

HU	Operational	0%	-1%	0%	-26%	0%	0%	-26%
HU	Total	-3%	-4%	-1%	-16%	-1%	0%	-24%
IE	Embodied	-12%	-13%	-13%	21%	-12%	-5%	-22%
IE	Operational	-1%	0%	0%	-29%	0%	0%	-29%
IE	Total	-3%	-2%	-2%	-20%	-2%	-1%	-28%
IT	Embodied	-21%	-25%	-4%	41%	-9%	-2%	1%
IT	Operational	0%	-1%	0%	-34%	0%	0%	-34%
IT	Total	-3%	-5%	-1%	-21%	-2%	0%	-28%
LT	Embodied	-15%	-9%	-8%	26%	-4%	-6%	-1%
LT	Operational	0%	-1%	0%	-34%	0%	0%	-34%
LT	Total	-7%	-4%	-4%	-4%	-2%	-3%	-18%
LU	Embodied	-17%	-24%	-19%	8%	-15%	-3%	-44%
LU	Operational	1%	-1%	0%	-38%	0%	0%	-36%
LU	Total	-4%	-7%	-5%	-26%	-4%	-1%	-38%
LV	Embodied	-13%	-11%	-11%	28%	-4%	-7%	1%
LV	Operational	-1%	-1%	0%	-36%	0%	0%	-36%
LV	Total	-4%	-3%	-3%	-19%	-1%	-2%	-27%
MT	Embodied	-18%	-13%	-10%	22%	-7%	-1%	-20%
MT	Operational	-1%	-1%	0%	-40%	0%	0%	-41%
MT	Total	-8%	-6%	-4%	-13%	-3%	-1%	-32%
NL	Embodied	-25%	-24%	-8%	30%	-24%	-3%	-36%
NL	Operational	-1%	-1%	0%	-40%	0%	0%	-40%
NL	Total	-7%	-7%	-2%	-21%	-6%	-1%	-39%
PL	Embodied	-20%	-25%	-7%	22%	-13%	-2%	-29%
PL	Operational	0%	-3%	0%	-43%	0%	0%	-43%
PL	Total	-3%	-7%	-1%	-31%	-2%	0%	-41%
PT	Embodied	-26%	-21%	-6%	26%	-10%	-2%	-16%
PT	Operational	-1%	-1%	0%	-32%	0%	0%	-32%
PT	Total	-13%	-11%	-3%	-4%	-5%	-1%	-24%
RO	Embodied	-12%	-12%	-11%	15%	-12%	-2%	-24%
RO	Operational	-1%	-1%	0%	-29%	0%	0%	-30%
RO	Total	-4%	-4%	-3%	-17%	-3%	-1%	-28%
SE	Embodied	-20%	-43%	-8%	27%	-22%	-3%	-34%
SE	Operational	-2%	-6%	0%	-48%	0%	0%	-48%
SE	Total	-14%	-31%	-6%	3%	-15%	-2%	-39%
SI	Embodied	-20%	-16%	-11%	20%	-14%	-2%	-23%
SI	Operational	1%	-1%	0%	-25%	0%	0%	-25%
SI	Total	-5%	-6%	-3%	-12%	-4%	-1%	-25%
SK	Embodied	-15%	-14%	-10%	19%	-14%	-3%	-22%
SK	Operational	1%	0%	0%	-44%	0%	0%	-44%
SK	Total	-2%	-3%	-2%	-32%	-3%	-1%	-40%

Supplementary Table 9. Useful floor area of buildings demolished, newly build, renovated or in stock, for each scenario in 2020, 2030, 2040 and 2050.

Scenario	Area (Mm^2)	2020	2030	2040	2050
BAU (Business-As-Usual)	Demolished	63	81	91	98
	New	315	275	262	240

	Renovated	306	327	345	360
	Stock	30,921	33,007	34,821	36,377
HOPE (Honoring Official Policy Expectations)	Demolished	63	65	36	13
	New	315	257	205	161
	Renovated	306	1,199	1,064	137
	Stock	30,921	33,004	34,802	36,362
COPE (Capacity-Orchestrated Policy Execution)	Demolished	63	73	53	25
	New	315	266	223	170
	Renovated	306	672	1,060	834
	Stock	30,921	33,005	34,811	36,363
SMART (Strategy Mix Approach for Robust Trajectories)	Demolished	63	44	21	6
	New	315	115	91	72
	Renovated	306	685	1,030	891
	Stock	30,921	31,730	32,414	33,045

Supplementary References

1. Alaux, N. *et al.* Whole-life greenhouse gas emission reduction and removal strategies for buildings: Impacts and diffusion potentials across EU Member States. *J. Environ. Manage.* **370**, 122915 (2024).

2. Röck, M. *et al.* wlc-scenario-explorer: An open source tool for exploring whole life cycle emission scenarios of transnational building stocks. doi:10.5281/ZENODO.13315281.

3. Urban, P., Karlsson, I. & Nipius, L. Policies to reduce whole-life carbon in the built environment. Learnings from the EU and Sweden. *CEPS IN-DEPTH ANALYSIS* (2025).

4. Mastrucci, A., van Ruijven, B., Byers, E., Poblete-Cazenave, M. & Pachauri, S. Global scenarios of residential heating and cooling energy demand and CO2 emissions. *Clim. Change* **168**, 1–26 (2021).

5. Mastrucci, A., Guo, F., Zhong, X., Maczek, F. & van Ruijven, B. Circular strategies for building sector decarbonization in China: A scenario analysis. *J. Ind. Ecol.* **28**, 1089–1102 (2024).

6. Alaux, N., Schwark, B., Hörmann, M., Ruschi Mendes Saade, M. & Passer, A. Assessing the prospective environmental impacts and circularity potentials of building stocks: An open-source model from Austria (PULSE-AT). *J. Ind. Ecol.* **28**, 1435–1448 (2024).
