# Supplementary material for: Context-specific life cycle emissions pathways for EU buildings and construction
Source: Nat Commun. 2026 May 25;17:6804. doi: 10.1038/s41467-026-73433-1 (PMC13385351; doi:10.1038/s41467-026-73433-1)
Supplement: Supplementary file 2 — Reporting Summary [file 41467_2026_73433_MOESM2_ESM.pdf]

nature portfolio

Corresponding author(s): Alexander Passer

Last updated by author(s): Apr 13, 2026

Reporting Summary

Nature Portfolio wishes to improve the reproducibility of the work that we publish. This form provides structure for consistency and transparency in reporting. For further information on Nature Portfolio policies, see our Editorial Policies and the Editorial Policy Checklist.

Statistics

For all statistical analyses, confirm that the following items are present in the figure legend, table legend, main text, or Methods section.

n/a	Confirmed	
☒	☐	The exact sample size (*n*) for each experimental group/condition, given as a discrete number and unit of measurement
☒	☐	A statement on whether measurements were taken from distinct samples or whether the same sample was measured repeatedly
☒	☐	The statistical test(s) used AND whether they are one- or two-sided<br>*Only common tests should be described solely by name; describe more complex techniques in the Methods section.*
☒	☐	A description of all covariates tested
☒	☐	A description of any assumptions or corrections, such as tests of normality and adjustment for multiple comparisons
☒	☐	A full description of the statistical parameters including central tendency (e.g. means) or other basic estimates (e.g. regression coefficient) AND variation (e.g. standard deviation) or associated estimates of uncertainty (e.g. confidence intervals)
☒	☐	For null hypothesis testing, the test statistic (e.g. *F*, *t*, *r*) with confidence intervals, effect sizes, degrees of freedom and *P* value noted<br>*Give P values as exact values whenever suitable.*
☒	☐	For Bayesian analysis, information on the choice of priors and Markov chain Monte Carlo settings
☒	☐	For hierarchical and complex designs, identification of the appropriate level for tests and full reporting of outcomes
☒	☐	Estimates of effect sizes (e.g. Cohen's *d*, Pearson's *r*), indicating how they were calculated

Our web collection on statistics for biologists contains articles on many of the points above.

Software and code

Policy information about availability of computer code

Data collection	The data was processed using the MMG tool, the open-source SLiCE data format [https://doi.org/10.1016/j.spc.2024.01.005] and the PULSE-EU model v1.0.0 [https://doi.org/10.5281/zenodo.19550009].
Data analysis	Data analysis was performed in python using the following packages: matplotlib=3.10.8, plotly=6.3.0, python-kaleido=1.2.0, polars=1.39.3, pandas=3.0.2, geopandas=1.1.3, shapely=2.1.2, geos=3.14.1 and openpyxl=3.1.5.

For manuscripts utilizing custom algorithms or software that are central to the research but not yet described in published literature, software must be made available to editors and reviewers. We strongly encourage code deposition in a community repository (e.g. GitHub). See the Nature Portfolio guidelines for submitting code & software for further information.

Data

Policy information about availability of data

All manuscripts must include a data availability statement. This statement should provide the following information, where applicable:

- Accession codes, unique identifiers, or web links for publicly available datasets
- A description of any restrictions on data availability
- For clinical datasets or third party data, please ensure that the statement adheres to our policy

Data availability:
The minimum dataset required to interpret, verify and extend the results of this study comprises (i) the archetype-related datasets (life cycle scenario definitions,

inventories and impact assessment results), (ii) the scenario input data (the detailed list of greenhouse gas mitigation strategies, the input data used for their implementation and the Member State implementation capacities), and (iii) the scenario output data. Archetype-related data generated in this study have been deposited in the KU Leuven Research Data Repository [doi:10.48804/VXJUEW, doi:10.48804/JCTCAP and doi:10.48804/RUHLNF). These files are openly available under the CC BY 4.0 license. Scenario input data are available in a separate publication [https://doi.org/10.1016/j.jenvman.2024.122915] under CC BY-NC-ND 4.0 license, and as an archival snapshot of the PULSE-EU model inputs [https://doi.org/10.5281/zenodo.19550009]. Scenario output data generated in this study are can be interactively explored and downloaded via the WLC-Scenario-Explorer [https://doi.org/10.5281/zenodo.13315281] under the CC BY 4.0 license. Source data underlying the figures are provided in the Supplementary Information file of this article. Full life cycle impact assessment (LCIA) results at detailed resolution contain third-party content from the ecoinvent database and are therefore available under restricted access to comply with the ecoinvent End-User License Agreement. Access is restricted to researchers who hold a current ecoinvent license for version v3.6, cut-off and can be granted solely for the purpose of reproducing the findings of this study. To request access, contact the corresponding authors with (i) proof of a valid ecoinvent license, (ii) institutional affiliation, and (iii) a brief description of the intended use; approved requesters will be asked to agree to a non-redistribution and non-commercial use condition in line with the ecoinvent license. Upon approval, access will be provided via a secure link.

Code Availability
The PULSE-EU model, which is used to upscale the building archetypes to the stock level and projects the future scenarios, is open-source at https://github.com/ITE-NHB/PULSE-EU under the Graz University of Technology Software License Agreement, and is archived at https://doi.org/10.5281/zenodo.19550009.

Research involving human participants, their data, or biological material

Policy information about studies with human participants or human data. See also policy information about sex, gender (identity/presentation), and sexual orientation and race, ethnicity and racism.

Reporting on sex and gender	n/a
Reporting on race, ethnicity, or other socially relevant groupings	n/a
Population characteristics	n/a
Recruitment	n/a
Ethics oversight	n/a

Note that full information on the approval of the study protocol must also be provided in the manuscript.

Field-specific reporting

Please select the one below that is the best fit for your research. If you are not sure, read the appropriate sections before making your selection.

☐ Life sciences ☐ Behavioural & social sciences ☒ Ecological, evolutionary & environmental sciences

For a reference copy of the document with all sections, see nature.com/documents/nr-reporting-summary-flat.pdf

Ecological, evolutionary & environmental sciences study design

All studies must disclose on these points even when the disclosure is negative.

Study description	This study uses a comprehensive building stock model to analyze buildings and construction decarbonization pathways in the European Union (EU). It evaluates 4,096 different scenarios to determine the strategies required to meet the EU's 2030 and 2050 climate goals. The research compares the effectiveness of current policies focused on energy efficiency and materials against a more integrated approach that includes per-capita space demand reduction, use of bio-based materials and circular economy measures, all while considering national implementation capacities.
Research sample	This is a deterministic computational modeling study; no organisms or human participants were involved. The “sample” comprises modeled entities rather than experimental subjects: the building stocks of the 27 EU Member States, evaluated annually from 2020 to 2050, under a set set of 4,096 mitigation scenarios. No sub-sampling was performed and there were no biological manipulations. The population represented is the EU-27 building stock, representing about 31 billions square meters of useful floor area in 2020. This was compiled from established datasets: AmBIENCe [https://www.bpie.eu/wp-content/uploads/2022/02/AmBIENCe_D4.1_Database-of-grey-box-model-parameter-values-for-EU-building-typologies-update-version-2-submitted.pdf], Hotmaps [https://www.hotmaps-project.eu/wp-content/uploads/2020/09/brochure-hotmaps-2020-web.pdf] and the Buiding Stock Observatory [https://building-stock-observatory.energy.ec.europa.eu/factsheets/].
Sampling strategy	No statistical sample size calculation or analysis was performed because results are not based on statistical sampling. The analysis size is fixed by design: 27 countries × 4,096 scenario combinations × 31 annual time steps. This is sufficient because it (i) covers the entire EU-27 population relevant to the research question and (ii) exhaustively enumerates the defined scenario space within documented national implementation capacities.
Data collection	Data collection consisted of assembling and curating secondary datasets and defining model inputs. Data collection include the archetype-related datasets (life cycle scenario definitions, inventories and impact assessment results), and the scenario input data

	(the detailed list of greenhouse gas mitigation strategies, the input data used for their implementation and the Member State implementation capacities), all of which are specified in the Data Availability statement and Methods section of the article.
Timing and spatial scale	Model input datasets (archetypes, scenario parameters, and country-specific implementation capacities) were compiled by the authors between 01/2023 and 06/2025. The modeling horizon spans 2020–2050 with annual time steps. Spatial scale: national building stocks for the 27 EU Member States, with results reported at country level and aggregated as EU-27. Buildings of each Member State are not modeled individually but grouped into building archetypes. A total of 15,026 archetypes were created for the entire EU building stock. More details on country-specific building archetypes are available in the KU Leuven Research Data Repository [doi:10.48804/VXJUEW, doi:10.48804/JCTCAP and doi:10.48804/RUHLNF).
Data exclusions	No data exclusions were necessary for the analysis.
Reproducibility	This is a deterministic computational modelling study; given identical inputs and software versions, the workflow produces identical outputs. To verify reproducibility, we (i) version-controlled the PULSE-EU model code and inputs; (ii) specified the computational environment (environment.yml); (iii) archived exact snapshots of code and data with DOIs (see Code/Data Availability); and (iv) re-executed the workflow from a clean environment. Reproduction requires access to the licensed ecoinvent database (version 3.6).
Randomization	This was not relevant to our study.
Blinding	Blinding was not applicable. This study did not involve human or animal subjects, experimental treatments, or allocation to groups.

Did the study involve field work? ☐ Yes ☒ No

Reporting for specific materials, systems and methods

We require information from authors about some types of materials, experimental systems and methods used in many studies. Here, indicate whether each material, system or method listed is relevant to your study. If you are not sure if a list item applies to your research, read the appropriate section before selecting a response.

Materials & experimental systems

n/a	Involved in the study	
☒	☐	Antibodies
☒	☐	Eukaryotic cell lines
☒	☐	Palaeontology and archaeology
☒	☐	Animals and other organisms
☒	☐	Clinical data
☒	☐	Dual use research of concern
☒	☐	Plants

Methods

n/a	Involved in the study	
☒	☐	ChIP-seq
☒	☐	Flow cytometry
☒	☐	MRI-based neuroimaging

Plants

Seed stocks	n/a
Novel plant genotypes	n/a
Authentication	n/a
