# Supplementary material for: Context-specific life cycle emissions pathways for EU buildings and construction
Source: Nat Commun. 2026 May 25;17:6804. doi: 10.1038/s41467-026-73433-1 (PMC13385351; doi:10.1038/s41467-026-73433-1)
Supplement: Supplementary file 3 — Transparent Peer Review file [file 41467_2026_73433_MOESM3_ESM.pdf]

nature portfolio

Peer Review File

Context-specific life cycle emissions pathways for EU buildings and construction

Corresponding Author: Professor Alexander Passer

This file contains all reviewer reports in order by version, followed by all author rebuttals in order by version.

Version 0:

Reviewer comments:

Reviewer #1

(Remarks to the Author)
The authors attempt to address a very interesting topic with an effort to cover EU-27 that is relevant for international readers and policy makers. At first, the paper reads as a black box analysis. The method is revealed at the end but still it does not provide sufficient background information to inform the reader about the assumptions, calculation approach, scenario analysis etc, in order to be able to adopt-repeat or adapt the method.
There is no convincing evidence that the model has been properly validated before proceeding with the analysis that will also give confidence to the results.

The authors need to restructure their paper and enhance its contents, with more information on the approach and the tools (e.g. assumptions, inputs), provide sufficient evidence to validate that the proposed method/model, discuss the results going beyond observations and reiteration of data from the visualized results.

Given the extend of the necessary changes/enhancements/write-up, this reviewer does not recommend a simple revision of this paper, but encourages the authors to prepare and resubmit a new paper.

Some comments that were noted during the review of the manuscript are given below for future consideration. The comments are not listed in terms of importance but rather as they were encountered in the text during this review.

L54 "sufficiency-related measures "? Not clear
L56-58 Not clear
L63 Diffusing the methodology in the introduction is not appropriate. Need a clear presentation of the methodology before section 2-Results

L72-74 How did the authors come up with this breakdown? Need to provide a reference

L80 What are the "current policy documents"?

L82 Where is this data coming from?

L82-84 What is the basis/justification of this statement?

L87-89 What is the basis/justification of this statement?

L91-98 The discussion of (possible) results or data from another study is very confusing. Not knowing and understanding the modeling approach, the assumptions, input data etc make it very difficult, if not impossible, to understand the basis of these statements or accept the derived conclusions.
The scenarios are vague

Figure 1 - Need a reference. Where is this data coming from? especially for this detailed level of analysis.
If the authors developed these figures then need to elaborate in much more detail, e.g. assumptions, tools used to model, what is accounted for etc. …. Apparently, at the end it is implied that these are results from the authors' work/analysis.

However, this is not evident to the reader.

The text on p. 5 ("...which can be activated individually using the WLC-Scenario-Explorer online tool16. ") may be providing some relevant information. However, this text needs to be moved earlier (before Fig 1) and most important one needs to elaborate more how this is done, how all the different strategies are taken into account, what assumptions are made, what is included in the scenarios (refer to section 3 in the supplementary material)

L104 "4,096 additional scenarios" Where are these scenarios elaborated?

Table 1 Where is this information coming from? Provide a reference
If the authors compiled this Table then need to elaborate the contents in more detail

L114 Expressing the materials-related embodied GHG emissions on a per capita basis is rather awkward. Although it is common to normalize operational emissions per capita since this is directly related to population, this may not be the case for the embodied emissions. The benchmarks expressed per capita are biased against small countries and favor large countries, although the amount of GHG emissions is the important parameter.
In addition, it is not evident where the specific "materials-related embodied emissions" data is coming from. Does the calculation approach consider cross boarder material flow? imported steel rods, tiles or other building construction materials from one country to another? This would actually be more representative for island countries like Cyprus and Malta that practically import materials and electricity in other cases like Luxembourg.
As a recommendation, consider normalizing against the new building construction and renovation floor area.

L118-123 Similar comments. Need to reveal the assumptions in order to better understand what is included in the calculations.

L122 What is the basis of this statement? As much as a cold climate drives heating demand, a hot climate would drive cooling demand and one can expect similar results if electricity generation is based on fossil-fuel power generation.
L122-123 Sweden also has a cold climate but follows a different pattern. The observations should be supported by proper elaboration.

L129-130 This explains the lower operational emissions per capita in Sweden.

L138-139 In general, this is a valid statement, although one needs to first know the national constraints and implementation capacities. Can the authors provide an overview of what needs to be accounted? In addition, the authors should expand and provide some recommendations for the different approaches. For example, present the specific characteristics of the scenarios as they apply them to the MS.

L156-158 "Operational emission improvements ..." This is an important statement and needs to be better elaborated.

L161-162 Not clear

L162 "(ii) Low-carbon ..." Why ?

L165-166 Why?
The different concepts are not clear

L169-176 Interesting results but there is a need to substantiate them. What are some critical national factors/characteristics that justify/explain these differences?
Need to understand what is the basis for these differentiations since policies change over time and countries need to learn from each other.

Section 4 - Methodology is a very important section to come at the end. If this is intentional, then the authors must at least provide sufficient information in the earlier sections to address the issues and concerns that were included in the previous sections. In addition, clear references to section 4 must be made in the earlier sections of the paper, in order to properly direct the reader to where the necessary details are revealed.

Section 4.1 It comes as a great surprise that the authors do not mention TABULA in their review of building typology concepts.

L259 "Primary data sources were existing EU projects and studies31,32 " This is very abstract. What does this refer to? Elaborate the specific primary data. Discuss 31 & 32

L261-262 "assuming the same building geometry 261 and materialization of the latest building age class. " Not clear

L262-265 What are the three energy standards?
How are the HVAC systems adapted to the energy performance requirements in the respective MS? What are these requirements in each MS? Where and how is the gas phase out in each MS?

It is not clear whether the authors have validated the model of the building stock model that was set up.

L280-285 What are the specifics?

L288-289 Vague statement, need to be more specific

L290-291 The rates remain unknown. Considering the scarcity of this information, the reader is left wondering what national literature was considered, for what countries, and what was the outcome.

L291-296 The outline is reasonable but it is missing the necessary details to properly understand the process, assumptions and differences at national level.

Section 4.3 is very abstract.

L342-344 Not clear if these changes are applied at the same rate for all MS

(Remarks on code availability)

Reviewer #2

(Remarks to the Author)
The title requires improvement in terms of English clarity. Moreover, the use of the term “risk” is not substantiated throughout the manuscript. No definition of “risks” is provided, and its relevance to the study is unclear. Please revise the title accordingly.

The title of Section 2.2 currently uses a verb form. It is recommended to avoid verbs in section titles for consistency and clarity.

You claim that the defined archetypes align with the classification used in the Building Stock Observatory (BSO), describing it as the most advanced and up-to-date framework in the EU. This statement requires further justification. The BSO is known to be incomplete and missing important information in certain areas. Please explain in more detail how the archetypes were retrieved from BSO and how this alignment was validated.

The choice of using a 50-year reference study period for assessing archetypes should be justified. Furthermore, clarification is required for the case of existing buildings constructed in the 1970s, 1980s, and 1990s. How was this applied, given that the analysis is reported for 2030, 2040, and 2050?

The PULSE EU model was originally developed for Austria. Please clarify how this model was adapted to other Member States, and what modifications or assumptions were introduced.

Was the Level(s) framework considered in your study? If so, please comment on how it relates to your methodology. If not, please explain why it was excluded.

(Remarks on code availability)
Please clarify whether the defined archetypes are accessible to the public or research community, and in what format they can be obtained.

Reviewer #3

(Remarks to the Author)
The manuscript investigates the necessary GHG emission reduction in the building stock of the European Union Member States to achieve the collective GHG emissions target by 2030 and beyond. The topic is of interest, and the research has potential. However, several underlying data and methodological assumptions are missing, making it challenging to understand the results. Moreover, the discussion is too broad and does not provide actionable insights and tailored takeaways based on the results.

Major comments:
- A list of policies and measures considered under each strategy, along with their potential embodied and operational carbon savings, is missing. Are these measures based on national plans submitted to the European Commission, or are they assumed based on the target?
- Figure 3 presents the impact of assumed strategies, but the specific measures are not clear. For example, what does ‘implementation of improved materials’ mean? Although more information is provided in the supplementary material (Table S2), it is still not clear enough.
- The building renovation rates and depths assumed for each scenario are unclear. You mention light, medium, and deep levels, but it is not clear how each level is included in each scenario. The HOPE scenario assumes an accelerated renovation wave, but what does this mean? Is it related to the Renovation Wave strategy goals? How many buildings (or

floor area) are renovated by 2030, 2040, and 2050 in each scenario?
- Which implementation constraints are considered in the COPE scenario, and how are they addressed in the SMART scenarios?
- Are country-specific targets and measures to decarbonize the building stock and achieve EU-wide goals considered in the analysis?
- Besides 'reduce space demand per capita' which other sufficiency measures are considered in the analysis?
- The discussion remains too general and fails to provide country and strategy -specific insights and examples. Statements such as 'reducing operational emissions through thermal renovation and exchange of heating systems remains the highest-impact lever, and must be systematically complemented by measures that mitigate embodied emissions, including material decarbonization, circular economy principles, and sufficiency' are overly broad and generic, do not offer innovative insights, and makes it difficult to understand it in practice.

Minor comments
- Embodied carbon represents the major share of a building's life cycle carbon in new buildings; In existing EU buildings, the high energy consumption for building operation is still high and therefore generates high operational carbon.
- Figure 3 is very difficult to read.
- Figure S4, please provide the countries grouping in the four climatic zones.
- What does CRRS stands for in Table S2?

(Remarks on code availability)

Version 1:

Reviewer comments:

Reviewer #2

(Remarks to the Author)
All major comments and concerns were adequately addressed.

(Remarks on code availability)

Reviewer #4

(Remarks to the Author)
In response to Reviewer #3, the manuscript has clearly improved in terms of methodological transparency and the explicit description of strategies, renovation dynamics, and implementation constraints. The clarification of scenario activation logic (HOPE, COPE, SMART), the addition of quantitative renovation rates, and the expanded explanation of strategy categories meaningfully address earlier concerns about missing assumptions. The discussion section has also been strengthened with more concrete examples and country illustrations.

That said, some elements of Reviewer #3's critique are only partially resolved. While strategy effectiveness is differentiated across Member States, the policy implications could benefit from a concise synthesis that explicitly articulates the implied prioritisation emerging from the results (e.g., what generally dominates EU-wide and under which country conditions alternative strategies become more effective).

In addition, contextualising the headline mitigation figures (8.53 $GtCO_2e$ and the 2.72 $GtCO_2e$ shortfall) with percentages or benchmarks would improve interpretability.

A brief expansion on the practical feasibility constraints of sufficiency measures—particularly political and behavioural barriers—would further strengthen credibility.

Finally, situating the results more explicitly within the current EU policy architecture, including ETS2 and potential financing mechanisms such as ETS2 revenues and the Social Climate Fund, would enhance policy completeness without requiring additional modelling.

Overall, Reviewer #3's main methodological concerns have been substantially addressed, though the clarity and strategic framing of the policy conclusions could still be sharpened.

(Remarks on code availability)

Open Access This Peer Review File is licensed under a Creative Commons Attribution 4.0 International License, which permits use, sharing, adaptation, distribution and reproduction in any medium or format, as long as you give appropriate credit to the original author(s) and the source, provide a link to the Creative Commons license, and indicate if changes were made.
In cases where reviewers are anonymous, credit should be given to 'Anonymous Referee' and the source.
The images or other third party material in this Peer Review File are included in the article's Creative Commons license, unless indicated otherwise in a credit line to the material. If material is not included in the article's Creative Commons license and your intended use is not permitted by statutory regulation or exceeds the permitted use, you will need to obtain permission directly from the copyright holder.
To view a copy of this license, visit https://creativecommons.org/licenses/by/4.0/

NCOMMS-25-61669-T: Answer to reviewers

Dear Reviewers,

Thank you for reviewing the paper. We appreciate the quality of the review and have revised the paper accordingly. Below is a detailed response to your comments. In bold you will find your original comment, in black indented you will find our response, and in blue indented you will find a copy of the text in the paper that incorporates your comment. The references to specific line numbers refer to the track-changed version of the article.

Comments from Reviewer 1

The authors attempt to address a very interesting topic with an effort to cover EU-27 that is relevant for international readers and policy makers. At first, the paper reads as a black box analysis. The method is revealed at the end but still it does not provide sufficient background information to inform the reader about the assumptions, calculation approach, scenario analysis etc, in order to be able to adopt-repeat or adapt the method. There is no convincing evidence that the model has been properly validated before proceeding with the analysis that will also give confidence to the results. The authors need to restructure their paper and enhance its contents, with more information on the approach and the tools (e.g. assumptions, inputs), provide sufficient evidence to validate that the proposed method/model, discuss the results going beyond observations and reiteration of data from the visualized results. Given the extend of the necessary changes/enhancements/write-up, this reviewer does not recommend a simple revision of this paper, but encourages the authors to prepare and resubmit a new paper. Some comments that were noted during the review of the manuscript are given below for future consideration. The comments are not listed in terms of importance but rather as they were encountered in the text during this review.

> Thank you for your thoughtful comments and for recognizing the relevance of our study. We have carefully considered each of your suggestions and made extensive revisions accordingly. Specifically, we have:
>
> - Thoroughly revised the Methods section and added clear evidence of model validation.
> - Reworked the manuscript to improve flow and readability.
> - Enhanced the data visualizations and expanded the discussion of our results.
>
> We hope these changes meet your expectations and address the points you raised. We appreciate the time you have invested in reviewing our work and look forward to receiving any further feedback on this revised version.

L54 "sufficiency-related measures "? Not clear. L56-58 Not clear.

> Thank you for your comment. We have rewritten the passage and clarified our meaning for “sufficiency measures” (see 56–63). We hope the revision makes the point much clearer.
>
> This disconnect is particularly pronounced for sufficiency measures or demand-side strategies, such as the reduction of the average living area per capita. These strategies aim at reducing energy and material consumption, but they remain poorly quantified, especially from an embodied emissions perspective. Given their potential for early adoption and the minimal economic or technical barriers involved, these could provide immediate and substantial GHG emission reductions while capital-intensive, low-carbon technologies continue to mature and scale[15].

L63 Diffusing the methodology in the introduction is not appropriate. Need a clear presentation of the methodology before section 2-Results.

Thank you very much for your thoughtful comment. We recognize that placing the methods section at the end of the manuscript can be confusing for readers. This layout follows the journal's author guidelines, which require the methods to appear after the results. To improve clarity, we have added a brief methodological overview at the end of the introduction (see lines 64-90) and explicitly described the article's structure. In addition, the methods section itself has been expanded to incorporate all of the details you highlighted in your other remarks. We hope these revisions make the manuscript easier to follow.

In this paper a comprehensive analysis of the life cycle GHG emissions of the EU building stock from 2020 to 2050 is presented, using a bottom-up, archetype-based model that integrates life cycle assessment and material flow analysis across all 27 MS. Building archetypes are defined at the MS level for residential and non-residential buildings for existing buildings (4,490 in total), new constructions (5,919), as well as renovations (4,617) to model future building stock activities and renovation intensities (light, medium, deep). For each archetype, detailed life cycle inventories are built hierarchically, from whole building to components to materials, using data from sources such as AmBIENCe[16], Hotmaps[17] and the Building Stock Observatory[18], linked to the ecoinvent[19] database and validated by regional experts. Life cycle impact assessment follows the MMG-SLiCE model[20] focusing on the global warming potential (total). The PULSE-EU building stock model up-scales archetype data to a 2020 baseline and projects scenarios to 2050. Calibration aligns new building permits and operational emissions with observed data, while validation against independent models (MESSAGEix-Buildings, STURM)[21,22] confirms credibility.

The projected scenarios are measure-driven, based on the implementation of GHG emission reduction strategies[14]. Six groups of strategies (circularity, reduced space demand, bio-based materials, operational emissions reduction, improved material production, as well as transport and construction processes) are parameterized for low, medium, and high ambition levels across 2020-2050, enabling scenario analyses that test alignment with EU policy targets. Each of these strategies can be activated individually using the WLC-Scenario-Explorer online tool[23]. A key component of the methodology is the integration of each MS implementation capacity, which is assessed against a broad set of national conditions. These include resource and industrial capacity (e.g., bio-based material availability, recycling rates), geographic and building stock characteristics (e.g., renovation potential, seismic stability), and the prevailing socio-economic and policy landscapes (e.g., national policy frameworks, workforce skills, social conditions)[14]. By incorporating these realistic national constraints, as a low, medium or high potential to implement each strategy, our analysis provides detailed projections that reconcile EU climate targets with implementation realities, offering a robust basis for policy design. The results of the analysis are presented in Sections 2 and discussed in Section 3, while the methodology is fully described in Section 4.

L72-74 How did the authors come up with this breakdown? Need to provide a reference

Thank you for your comment. The results presented are derived from our own modelling work. To improve clarity, we have added a brief statement and included a reference to the Methods section, where the approach is described in greater detail (see lines 93-96).

EU buildings emitted 808 MtCO_2e in 2020 (including fossil, biogenic, land use and land use change emissions) due to their construction (18%), maintenance (5%), operation (73%),

renovation (3%) and demolition (1%). These results are based on the upscaling of building archetype data to establish a baseline year in 2020 for the EU building stock (see Section 4.3).

L80 What are the "current policy documents"?

Thank you for the opportunity to clarify this point. We have rephrased the sentence and added a link to the supplementary information, which now includes a table listing the policy documents and targets that were considered (see lines 105-107).

Assuming that the targets formulated in current EU policies, such as the Energy Performance of Buildings Directive, are fully reached, by implementing the strategies expressed in these policy documents (HOPE, see table S7 in the supplementary information), …

L82 Where is this data coming from?

Thank you for your comment. As with the two preceding points, this data originates from our own analysis and modelling work. We have slightly rephrased the sentence to make it clearer that this is a finding of our study (see lines 107-109).

We find that the contribution of buildings to achieving the EU climate targets would imply a cumulative GHG emissions reduction of 8.53 $GtCO_2e$ from 2020 to 2050.

L87-89 What is the basis/justification of this statement?

Thank you for your comment. This result also stems from our own analysis, and we have now indicated it explicitly in lines 114-117.

This presents a risk to the achievement of EU climate targets. However, a large share of this increase in emissions (2.19 $GtCO_2e$ or 80%) could be compensated by activating a wider range of strategies beyond the ones expressed in policy documents (SMART), while still operating within realistic MS constraints (Figure S6), as the result of our analysis demonstrates.

L91-98 The discussion of (possible) results or data from another study is very confusing. Not knowing and understanding the modeling approach, the assumptions, input data etc make it very difficult, if not impossible, to understand the basis of these statements or accept the derived conclusions. The scenarios are vague.

Thank you for highlighting the need for greater transparency. In response, we have taken several steps to address your concerns:

- The Methods section now contains a comprehensive set of details, reflecting the suggestions you made throughout your review. The tracked-changes version of the manuscript shows that this portion has been thoroughly revised.
- Section 4 of the Supplementary Information has been updated to present additional quantitative data on the scenarios, providing a more concrete foundation alongside the revised Methods section.
- We have added a reference in the Data-Availability statement directing readers to the GitHub repository where the full suite of quantitative scenario data, including diffusion rates and projected GHG emission reduction potentials, is publicly available.

These revisions aim to ensure full transparency and reproducibility of our work.

Figure 1 - Need a reference. Where is this data coming from? especially for this detailed level of analysis. If the authors developed these figures then need to elaborate in much more detail, e.g.

assumptions, tools used to model, what is accounted for etc. …. Apparently, at the end it is implied that these are results from the authors' work/analysis. However, this is not evident to the reader.

Thank you for your comment. Indeed, the results stem from our own work, and, as required by the journal, the Methods section appears at the end of the article. Following your suggestion, we have inserted cross-references to the Methods section throughout the manuscript to make the connection clearer. In addition, we have added a descriptive caption to the title of Figure 1 to further guide the reader:

These results are based on the upscaling of building archetype data to the EU building stock and the projection of future scenarios, as explained in Section 4.3 and 4.5.

The text on p. 5 ("...which can be activated individually using the WLC-Scenario-Explorer online tool16. ") may be providing some relevant information. However, this text needs to be moved earlier (before Fig 1) and most important one needs to elaborate more how this is done, how all the different strategies are taken into account, what assumptions are made, what is included in the scenarios (refer to section 3 in the supplementary material).

Thank you for noticing the suggestion. We have introduced the WLC-Scenario-Explorer tool earlier in the text, at the end of the introduction, as you can see lines 76-82. Regarding the explanation of the methodological details and assumptions, we hope this is now clarified based on our replies to your other comments.

The projected scenarios are measure-driven, based on the implementation of GHG emission reduction strategies[14]. Six groups of strategies (circularity, reduced space demand, bio-based materials, operational emissions reduction, improved material production, as well as transport and construction processes) are parameterized for low, medium, and high ambition levels across 2020-2050, enabling scenario analyses that test alignment with EU policy targets. Each of these strategies can be activated individually using the WLC-Scenario-Explorer online tool[23].

L104 "4,096 additional scenarios" Where are these scenarios elaborated?

Thank you for your comment. The requested information is provided in Supplementary Information, Section 3 (see excerpt below). To help readers locate it more easily, we have added a reference to this section in the caption of Figure 1.

The modelling of future scenarios is measure-driven, meaning that it relies on the implementation of specific strategies to reduce the whole life GHG emissions of EU buildings. These strategies have been identified based on literature review and stakeholder consultation[2] and further grouped into six main categories or packages: (1) the implementation of circularity measures, (2) the reduction of per capita space demand, (3) the shift to low carbon, bio-based materials, (4) the reduction of operational emissions, (5) the improvement of material production processes and (6) the improvement of transport and construction processes. The link between the strategies from the original article[2] and this contribution is shown in Table S4. For each of these strategies, the capacity of each MS to implement them was also qualitatively assessed, allowing for consideration of local conditions within the scenario modelling, as shown in Figure S6. The scenarios are generated using four activation levels for each of the six strategies: (1) strategy not activated, (2) strategy activated to half the capacity of the MS, (3) strategy activated to the full capacity of the MS and (4) strategy activated to full capacity assuming all MS have a high capacity. These scenarios can be activated individually using the WLC-Scenario-Explorer online tool[3].

Table 1 Where is this information coming from? Provide a reference. If the authors compiled this Table then need to elaborate the contents in more detail.

Thank you for your comment. This table provides a simple overview of the scenarios modelled in the study. More details are available in the supplementary information (Section 4). We have added this to the title of the table to make it clearer for the reader.

Table 1. Overview of the policy-relevant scenarios applied to the EU building stock. More details regarding the modelling of these scenarios are available in the supplementary information (Section 4).

L114 Expressing the materials-related embodied GHG emissions on a per capita basis is rather awkward. Although it is common to normalize operational emissions per capita since this is directly related to population, this may not be the case for the embodied emissions. The benchmarks expressed per capita are biased against small countries and favor large countries, although the amount of GHG emissions is the important parameter. In addition, it is not evident where the specific "materials-related embodied emissions" data is coming from. Does the calculation approach consider cross boarder material flow? imported steel rods, tiles or other building construction materials from one country to another? This would actually be more representative for island countries like Cyprus and Malta that practically import materials and electricity in other cases like Luxembourg. As a recommendation, consider normalizing against the new building construction and renovation floor area.

Thank you for the insightful suggestion on the choice of denominator for reporting embodied GHG emissions. While we do understand your point, we would argue that allocating the total embodied carbon of the building stock to the whole population provides a meaningful indicator of the average carbon burden per person and aligns with the widely used metric of Domestic Material Consumption per capita, which is reported by Eurostat and the United Nations Sustainable Development Goals framework.

We would also like to emphasize that embodied emissions arise not only from new construction or renovation but also from the ongoing maintenance and eventual demolition of existing buildings. Because these activities affect the entire building stock rather than just the newly added or renovated floor area, normalising the emissions solely by new or renovated area would not capture the full picture. For this reason, we believe that a per-capita normalisation remains the most appropriate choice for our study.

Regarding your second point on cross-border material flows, we relied on the ecoinvent database for the background data. Consequently, our analysis is confined to the material-consumption markets covered by ecoinvent, and we did not supplement it with additional information on imports and exports of materials among EU countries. This considers a limitation of our study which could be addressed in the future.

L118-123 Similar comments. Need to reveal the assumptions in order to better understand what is included in the calculations.

Thank you for your comment. We have now disclosed the assumptions and methodological details you requested in the Methods section, and we hope this clarifies the components of our calculations.

L122 What is the basis of this statement? As much as a cold climate drives heating demand, a hot climate would drive cooling demand and one can expect similar results if electricity generation is based on fossil-fuel power generation. L122-123 Sweden also has a cold climate but follows a

different pattern. The observations should be supported by proper elaboration. L129-130 This explains the lower operational emissions per capita in Sweden

Thank you for your insightful remarks. While it is true that cooling demand is rising, as our results also show, the reduction in space heating demand in southern countries is considerably larger than the increase in cooling demand observed in these regions, which explains why operational emissions still remain lower. For example, many traditional homes in Spain lack heating systems altogether. In the case of Sweden, the relatively modest heating demand can indeed be attributed to its low-carbon energy mix. We have incorporated these considerations into the manuscript (see 159-162).

For countries like Sweden, which also have a cold climate, this reflects higher energy efficiency of the buildings and a low-carbon energy mix. For Southern European countries (Spain, Portugal), although space cooling is higher than in other countries, the relative increase remains smaller than the decrease in space heating, ensuring overall low operational emissions per capita.

L138-139 In general, this is a valid statement, although one needs to first know the national constraints and implementation capacities. Can the authors provide an overview of what needs to be accounted? In addition, the authors should expand and provide some recommendations for the different approaches. For example, present the specific characteristics of the scenarios as they apply them to the MS.

Thank you for the suggestion. To answer the first part of you comment, we have added a short description of the main indicators that can be used for national constraints, as you can see lines 172-176, and the full list in the supplementary information (Table S5).

These include, as provided in Table S5 and further detailed in Alaux et al. (2024), resource and industrial capacity (e.g., bio-based material availability, recycling rates), geographic and building stock characteristics (e.g., renovation potential, seismic stability), and the prevailing socio-economic and policy landscapes (e.g., national policy frameworks, workforce skills, social conditions)[14].

Regarding the second part of your comment, because our modelling framework integrates many interacting elements, it is inherently challenging to isolate a single cause for the observed differences. Factors such as building-stock composition (vacancy rates, density, occupancy), the carbon intensity of the local energy mix (both heating fuels and electricity generation), material production and transport processes, climate-driven heating versus cooling needs, construction practices (material choices and efficiency), and the assumed mitigation potential of each strategy (low, medium, high) all contribute to the outcomes. While we have outlined several of these drivers in the manuscript, a more precise attribution is not feasible given the current level of detail.

Importantly, we view this complexity as a strength: it reflects the country-specific realities that policymakers need to consider when designing effective interventions, rather than encouraging a one-size-fits-all approach. Accordingly, we have expanded the Discussion section to emphasize the policy relevance of these nuanced, context-dependent findings, as suggested by Reviewer 3.

L156-158 "Operational emission improvements ..." This is an important statement and needs to be better elaborated.

Thank you for your suggestion. While we are not entirely certain which aspects you would like expanded, we have added additional detail to the paragraph in question. The revised text can be found in lines 197-207.

EU policy provides a solid framework for GHG emission reductions that target some of the most effective strategies, namely the reduction of operational emissions, especially via the energy efficiency targets of the EPBD and the EED, as well as the improvement of material production processes, with the emission reduction targets from the ETS. However, the analysis of strategy effectiveness across 27 MS reveals significant heterogeneity (Figure 3). Operational emission improvements include thermal renovations, exchanges in heating systems, an increased share of renewable energy in the electricity and district heating, as well as the reduction in temperature setpoints. Though most effective at the EU level, they only represent the optimal strategy for 19 MS (when considering life cycle emissions). In particular, the Czech Republic achieves the highest reduction (40%) due to elevated per capita operational emissions and a limited embodied emissions trade-off.

L161-162 Not clear.

Thank you for your comment. We have rephrased this sentence which was indeed not clear, as you can see lines 210-214.

High embodied emission countries per capita (e.g. Austria, Cyprus) with high construction rates benefit most from reductions in the per capita space demand, which can rapidly reduce the number of new constructions through better use of the available, existing building stock, while the reduction of operational emissions is slower to implement, with a trade-off in embodied emissions

L162 "(ii) Low-carbon ..." Why?

Thank you for your comment. We have now provided a justification for this claim, which can be found in lines 215-219.

Low-carbon energy mix countries with high living standards (e.g. Sweden) also favor reductions in per capita space demand, because these embodied emissions dominate their life cycle emissions while other strategies, such as reduction in operational emissions or the improvement of material production processes, might be less effective than in other countries, due to their current use of low-carbon energy.

L165-166 Why? The different concepts are not clear.

Thank you for your comment. We have expanded the description of the circularity strategies to specify precisely what is modelled, and we have incorporated this clarification into the manuscript (see lines 219-224).

Countries with balanced operational-to-embodied emission ratios (higher than 40-50%, including Portugal, Spain, Bulgaria, Denmark, Lithuania) achieve optimal results through circularity measures, which include the extension of the service life of buildings through renovation, vacancy reduction, efficient structural material use, as well as increased reuse and recycling. , This reflects the smaller influence of operational emissions and could indicate higher vacancy rates, demolition rates or a higher potential for reuse and recycling.

L169-176 Interesting results but there is a need to substantiate them. What are some critical national factors/characteristics that justify/explain these differences? Need to understand what is the basis

for these differentiations since policies change over time and countries need to learn from each other.

Thank you for highlighting this point. Because our modelling framework integrates many interacting elements, it is inherently challenging to isolate a single cause for the observed differences. Factors such as building-stock composition (vacancy rates, density, occupancy), the carbon intensity of the local energy mix (both heating fuels and electricity generation), material production and transport processes, climate-driven heating versus cooling needs, construction practices (material choices and efficiency), and the assumed mitigation potential of each strategy (low, medium, high) all contribute to the outcomes. While we have outlined several of these drivers in the manuscript, a more precise attribution is not feasible given the current level of detail.

Importantly, we view this complexity as a strength: it reflects the country-specific realities that policymakers need to consider when designing effective interventions, rather than encouraging a one-size-fits-all approach. Accordingly, we have expanded the Discussion section to emphasize the policy relevance of these nuanced, context-dependent findings, as suggested by Reviewer 3 (lines 269-342).

Reducing operational GHG emissions being the most effective lever to reduce life cycle emissions in the majority of the MS, operational emissions policies should translate the main EU-related policies (EPBD, EED and RED) into ambitious national actions. Key measures include adopting comprehensive National Building Renovation Plans that chart a pathway to a climate-neutral stock by 2050, enforcing Minimum Energy Performance Standards for non-residential buildings, and applying the Zero-Emission Building standard to all new construction while progressively incorporating whole-life carbon limits. Supporting instruments such as Energy Performance Certificates, Renovation Passports, and targeted financial incentives must be widely available[27]. The EED's "Energy Efficiency First" principle should guide planning, investment and permitting decisions, with the public sector leading by renovating at least 3 % of its floor area annually and prioritizing high-performance retrofits. Finally, the RED calls for accelerated deployment of on-site and neighborhood renewable generation, streamlined legal frameworks for energy communities, and the integration of digital demand-side flexibility to optimize renewable use in buildings and districts[28]. These policies must be complemented by measures that mitigate embodied emissions, and allow strategies to be deployed where they are most effective, thereby navigating implementation capacity constraints.

Sufficiency measures, which aim to reduce the absolute demand for energy and materials to operate within planetary boundaries, can induce profound systemic effects[29,30]. In our study, they include the reduction of per capita space demand (most effective in in Austria and Cyprus), the extension of the service life of buildings through renovation and vacancy reduction (included in the circularity measures, most effective in Portugal, Spain, Bulgaria, Denmark and Lithuania), as well as the reduction in temperature setpoints (part of the operational emission measures). Despite being underrepresented in policy compared to efficiency and renewable energy strategies[31], their importance is well-established. Previous studies have shown that sufficiency is crucial for achieving carbon neutrality and energy security in Europe, as it alleviates pressure on supply-side transformations[32]. Indeed, scenarios that exclude behavioral change are projected to miss GHG neutrality targets in the EU[33]. Furthermore, from an energy systems perspective, integrating sufficiency is highly advantageous, as a lower electricity demand reduces the need for costly supply-side investments and grid infrastructure[10]. Our results corroborate these findings, demonstrating

that incorporating sufficiency, alongside with other measures, creates a more balanced and feasible GHG emissions trajectory. However, the practical application of sufficiency faces considerable challenges, notably socio-political acceptance and potential rebound effects[34], necessitating fundamental reforms in both the content and the design process of building sector policies[29].

Sufficiency-oriented policies should prioritize the optimal use of the existing building stock to avoid upfront embodied GHG emissions associated with new construction, while simultaneously reducing operational emissions through higher occupancy and intensified use of floor space. Effective implementation requires coordinated measures such as establishing vacancy registers and applying vacancy taxes to incentivize the re-entry of under-occupied units into the market[35], streamlining permitting procedures for repurposing and retrofits, and offering density bonuses or zoning concessions to reward adaptive reuse[28]. Other examples include raising awareness, rethinking communication and designing effective financial motivation, such as a combination of different financial incentives that reward downsizing through flat swaps, or the introduction of minimum occupancy rates[36]. Public authorities and developers can further stimulate flexible, resilient design by launching competitions and pilot projects focused on adaptable concepts, and by creating neighborhood-level agencies that advise tenants on downsizing or relocation across life stages.

For countries showing high potential to reduce embodied GHG emissions by using bio-based materials or improving materials production processes, as well as reuse and recycling, such as the Estonia, Romania, Ireland or the Netherlands, policy measures should combine incentives, standards, and market-creation mechanisms. Governments can introduce tax credits or subsidies for the manufacturing and procurement of certified bio-based products, while mandating minimum bio-content thresholds in public-sector contracts. In parallel, maintaining and enhancing the carbon sink capacities in land-based systems that provide these bio-based products is crucial[37]. Updating building codes and material specifications to recognize and reward low-embodied emissions bio-based materials encourages their adoption in construction. In parallel, standards for circularity, such as material passports, product-level carbon footprints, and verified recycled-content labels, facilitate traceability and market confidence[28]. Supporting research-and-development programs that improve durability of bio-based polymers accelerates technological maturity. Finally, implementing extended producer responsibility schemes, take-back obligations, and incentives for industrial symbiosis creates economic drivers for systematic reuse, recycling, and urban-mining of bio-based and conventional materials, closing material loops and lowering overall embodied emissions[28].

Section 4 - Methodology is a very important section to come at the end. If this is intentional, then the authors must at least provide sufficient information in the earlier sections to address the issues and concerns that were included in the previous sections. In addition, clear references to section 4 must be made in the earlier sections of the paper, in order to properly direct the reader to where the necessary details are revealed.

Thank you for the comment. We recognize that placing the methods section at the end of the manuscript can be confusing for readers. This layout follows the journal's author guidelines, which require the methods to appear after the results. We have added clear references to the methodological details, either in section 4 or in the supplementary information, throughout the text, as you suggested, and we hope it makes the analysis clearer and more transparent.

Section 4.1 It comes as a great surprise that the authors do not mention TABULA in their review of building typology concepts.

Thank you for raising this important point. We appreciate your concern and would like to clarify that the primary data sources used in our study (AmBIENCe, Hotmaps, and the Building Stock Observatory) were developed after the TABULA project. These sources not only incorporated most of the TABULA data but also expanded the scope to include all EU MS and non-residential buildings. As a result, we consider these datasets to be more comprehensive and up-to-date than TABULA.

In a limited number of cases, we did supplement our data with TABULA to address specific gaps in residential building information when it was not already covered by the other sources. We have now clarified this in lines 385–386 and further expanded the section on data sources (as also addressed in our response to your subsequent comment).

Attributes relative to building geometry are first derived from AmBIENC[16], then complemented with data from the Cost-effectiveness studies[42] and TABULA/EPISCOPE[43].

L259 "Primary data sources were existing EU projects and studies31,32 " This is very abstract. What does this refer to? Elaborate the specific primary data. Discuss 31 & 32.

Thank you for your suggestion for improvement. We followed your suggestion and added a whole paragraph explaining in more details the data sources and the specific data which was retrieved from each data source. You can find the new paragraph in lines 381-394.

For existing buildings, the stock is represented by 66 archetypes per EU MS, resulting in a total of 1,782 archetypes. In addition, we include variants in technical systems, leading to 4,490 archetypes. Each archetype is then assigned information regarding its building geometry, material composition, as well as heating, ventilation and air conditioning systems (HVAC), called attributes. Attributes relative to building geometry are first derived from AmBIENCe[16], then complemented with data from the Cost-effectiveness studies[42] and TABULA/EPISCOPE[43]. Regarding material composition, Hotmaps[17] is used to derive the materials composing floors, walls, roofs and windows, and the construction technologies are then detailed in terms of layer thickness and performance properties using AmBIENCe[16]. In order to validate the retrieved information and fill data gaps, a data review by regional experts who were part of the EU-WLC project[44] was conducted. Finally, all attributes related to HVAC were derived from AmBIENCe[16]. The existing building stock is characterized by its total useful floor area, which is taken from the BSO[18], alongside with building stock occupancy data, for different building archetypes (shown in Figures S1 and S2 in the SI).

L261-262 "assuming the same building geometry 261 and materialization of the latest building age class. " Not clear.

Thank you for your comment. We have rephrased the sentence (see lines 396-398), and we hope that, together with the preceding paragraph describing the building-archetype attributes, the passage is now clearer.

A first set of 243 conventional new building archetypes is created assuming the same attributes for building geometry and material composition as the latest building age class (2011–2019).

L262-265 What are the three energy standards? How are the HVAC systems adapted to the energy performance requirements in the respective MS? What are these requirements in each MS? Where and how is the gas phase out in each MS?

Thank you for your valuable feedback. We have now included a detailed paragraph outlining the various energy performance standards considered, as well as the specific requirements

across MS. Regarding the phase-out of oil and gas heating systems in new buildings, this is addressed at the archetype level, reflecting the distinct requirements of each MS. At the stock level, however, the approach depends on the scenarios, which are further elaborated in Section 4.5. These aspects have been clarified in lines 398–409 of the revised text.

For each of the 243 new archetypes, three energy performance standards are specified. The first corresponds to the standard energy performance level, aligned with national nearly-zero energy building requirements as implemented by 2021. The second reflects an advanced energy performance, based on passive energy standards as defined per region in a recent study[45]. The third represents sub-standard performance, consistent with the energy characteristics of the latest building age class (2011–2019). HVAC systems are adapted to meet the energy performance requirements applicable in each MS, by means of adjustments to the system efficiency and the phase-out of gas- and oil-based space heating systems in new buildings. This was validated by regional experts who were part of the EU-WLC project[44]. Possible phase-out of gas- and oil-based space heating systems in each MS at the stock level depends on the projected scenarios (more information in Section 4.5).

It is not clear whether the authors have validated the model of the building stock model that was set up.

Thank you for your helpful comment. The validation of the building stock model and its input data is indeed a critical component of our analysis. To address this, we have added a dedicated subsection to the methodology that provides a more detailed description of the validation process. You can find the new material in lines 510-534.

4.4. Calibration and validation of the model

The building stock model and its data are both calibrated and validated to ensure representativeness. Calibration is performed for new building construction, thereby directly affecting upfront embodied emissions, and for operational emissions. Together, they represent a large majority of the life cycle emissions of the building stock in the baseline year (2020). In particular, new building constructions are calibrated for the baseline year to reach 80% of the delivered building permits[61] averaged between 2014 and 2023 as a reference value. This is meant to consider a 20% difference between the number of building permits and the actual number of buildings built (as not all permits are actually built). Regarding operational emissions (B6) a calibration for the baseline year (2020) is also performed, using reference emissions for 2020 from the BSO[18]. Direct emissions are overtaken as provided and, due to differences in modelling scope, only indirect emissions for HVAC are considered (other electricity uses are not included).

Validation of the model is performed in two steps. First, the archetype-related attributes underwent multiple validation rounds by regional experts who were part of the EU-WLC project[44], as mentioned in Sections 4.1 and 4.2. Additionally, we use an existing, peer-reviewed model to critically review and verify the validity of the modelling results obtained from the main modelling pipeline, for the baseline year and the BAU scenario. The established MESSAGEix-Buildings model, a bottom-up building sector model to assess energy, material demands, and GHG emissions of buildings at the regional and global scales under different socioeconomic, technological, climate and policy scenarios, as well as the STURM model, a stock turnover model based on dynamic material flow analysis to assess new constructions, demolitions and renovation activities, are deployed for generating those additional validation runs[21,22], as shown in the SI (Figures S7 and S8). The comparison demonstrates that the PULSE-EU produces results within a reasonable and credible range when benchmarked against an

independently developed, peer-reviewed framework. Minor discrepancies can be attributed to differences in data sources, modeling assumptions, and structural formulations between the two models.

L280-285 What are the specifics?

Thank you for your comment. We have now expanded the paragraph to provide additional detail on how the life cycle inventories are structured; you can see the revisions on lines 427-433.

For each of these archetypes, comprehensive life cycle inventories (LCI) are defined, with a hierarchical method for building decomposition[48]. More specifically, the modelling of buildings is structured in a hierarchical way from building archetype to building component (e.g. roof) to material level (e.g. concrete), using the material composition attributes that were defined for each archetype. These element definitions have undergone a review process by regional experts from the EU-WLC project[44] to maximize the representation of the heterogeneity of existing buildings and to validate uncertain information.

L288-289 Vague statement, need to be more specific. L290-291 The rates remain unknown. Considering the scarcity of this information, the reader is left wondering what national literature was considered, for what countries, and what was the outcome.

Thank you for your valuable comments. We have revised the paragraph to include additional information about the data sources. Specifically, we have now published these scenarios in an open-access research data repository. These updates can be seen in lines 327-336 of the manuscript.

The life cycle scenarios for transport, end-of-life waste treatments and reference service lives of building components are established at the MS level based on a literature review and expert consultation, which methodology is described in a published report[52]. the B-PCR[53] serves as a structure for these scenarios, in terms of building component and material classification. Scenarios for cleaning and maintenance during the use stage have been defined according to the Belgian MMG method[54]. The data and methodology behind the creating of these scenarios are openly available in a research data repository[55,56].

L291-296 The outline is reasonable but it is missing the necessary details to properly understand the process, assumptions and differences at national level.

Thank you for acknowledging that the outline is reasonable. We have added a graph (Figure S5) to the Supplementary Information. This figure illustrates the implications of the calculation for apartment buildings across all EU Member States, which should help clarify the methodological impact on national-level differences.

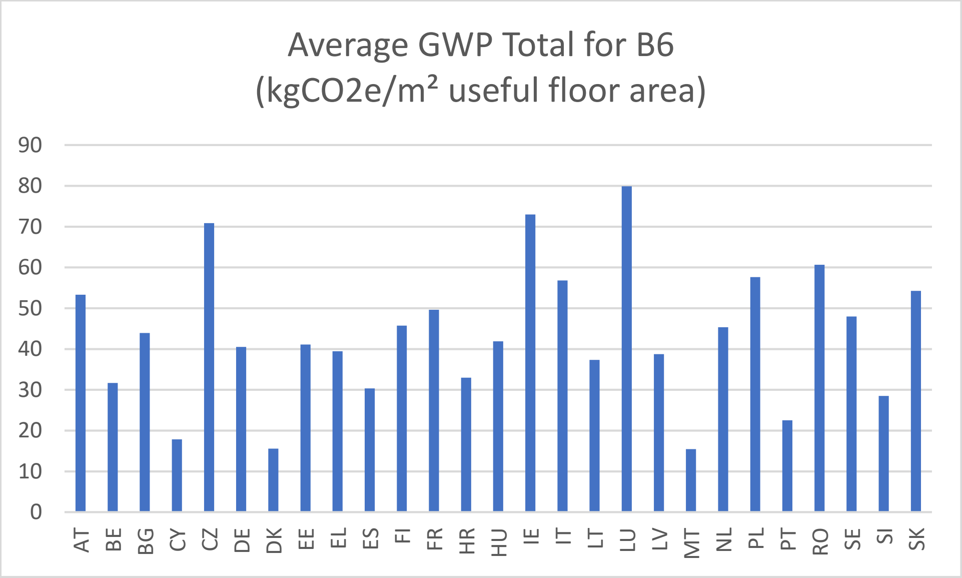

Section 4.3 is very abstract.

Thank you for your comment. We have undertaken a thorough revision of this section (see lines 467-509) and believe the changes now make the material clearer and more concrete.

The upscaling of building archetype data to establish a baseline year in 2020 for the building stock (see Figure S3 and S4 in the SI), along with the modelling of future scenarios for building stock development until 2050, is performed with the PULSE-EU building stock model (v1.0). This model is specifically developed to model the EU building stock, by expanding the foundational PULSE-AT model[59], originally developed for Austria, thereby extending its scope to cover all 27 EU MS. PULSE-EU projects future building stock activities, including new construction, maintenance, renovation, replacement and demolition, following the equations and upscaling the logic from the Austrian model, with these main adaptations: (i) Austrian-specific data is replaced with EU-specific data. An overview of the input data and sources is available on the GitHub publication of the model (see data availability statement). (ii) The model is adapted to import files generated with the SLiCE[20] building life cycle modelling structure, which affected the upscaling of in-use emissions (B2, B4, B6), as explained in Section 4.2. (iii) A new scenario modelling framework is created, which is explained in Section 4.5. (iv) The upscaling is performed based on useful floor area to align with the latest EPBD requirements[27], which is slightly different from the net floor area used in the Austrian model.

New residential building constructions are first estimated based on the expected evolution of the national population and average living area per capita[60]. This results in an amount of area that is needed for housing every year. It is assumed that the average living area per person corresponds to the total useful floor area of buildings in use divided by the total population of this specific year. This might cause differences to other models or data sources, if the considered area is different, or if the vacant stock is included in this area. Second, the capacity of the building stock to provide housing is calculated. This is performed by calculating the total useful floor area of residential buildings which are in use. This depends on the demolition and vacancy rate, and can thus be influenced by various scenario parameters. The difference between the demand for living area and the building stock capacity corresponds to the newly built area. For non-residential buildings, national population data are also used as a proxy indicator. However, to account for additional drivers influencing non-residential building construction dynamics, a calibration factor derived from historical construction rates is

introduced. The number of buildings constructed is then multiplied by the A1-A5 emissions provided by the MMG-SLiCE model.

Demolition activities are calculated using Weibull probability functions[21,22]. Each year, these functions provide a proportion of building archetypes that are demolished, based on their typology and age. All of the building archetypes that have not undergone a medium or deep renovation can be demolished. This would otherwise be considered unlikely within the time frame. The number of demolished buildings is then multiplied by the C1-C4 emissions provided by the MMG-SLiCE model. Renovations are implemented using the renovation rates specified in the scenarios. The number of renovated buildings is then multiplied by the B5 emissions provided by the MMG-SLiCE model. As explained in Section 4.2, emissions from maintenance, replacement and operational energy use are averaged across the reference study period of the building. At the stock level, the annual maintenance, replacement and energy use are calculated by the model, assuming that, for every year, these operations are needed for occupied or secondary buildings (empty dwellings are not considered for these annual emissions). Both the building stock activities and emission data are influenced by scenarios including GHG emission reduction strategies, the implementation of which is further detailed in Section 4.5.

L342-344 Not clear if these changes are applied at the same rate for all MS.

Thank you for your comment. We agree that this point required clarification. These changes are applied to the aggregated EU emissions and energy consumption, but not uniformly across all MS. This approach allows some MS to reduce emissions more quickly (if they have greater capacity), while still ensuring the overall EU target is achieved. We have added a clarification in the manuscript, lines 580-584.

When generating the scenarios, especially the HOPE scenario, the relevant strategies to reach these policy targets were gradually activated until the targets were reached. The total GHG emissions or energy consumption of the EU were compared to these EU policy targets, but not the ones from the MS. This allows, for example, for MS with higher capacity to reduce emissions faster than others, while still ensuring the overall EU target is achieved.

Comments from Review 2

The title requires improvement in terms of English clarity. Moreover, the use of the term "risk" is not substantiated throughout the manuscript. No definition of "risks" is provided, and its relevance to the study is unclear. Please revise the title accordingly.

Thank you for your insightful comment. Crafting a concise ten-word title that fully captures the study's message while maintaining clear English can indeed be challenging. In response, we have now completely revised the title, which we believe more accurately conveys the intended meaning of our study.

New title: Unlocking life-cycle emission reductions for EU buildings through policy flexibility

The title of Section 2.2 currently uses a verb form. It is recommended to avoid verbs in section titles for consistency and clarity.

Thank you for bringing this to our attention. Following your suggestion, we have revised the heading of Section 2.2 to eliminate the verb form.

2.2. EU climate policy in light of national constraints

You claim that the defined archetypes align with the classification used in the Building Stock Observatory (BSO), describing it as the most advanced and up-to-date framework in the EU. This statement requires further justification. The BSO is known to be incomplete and missing important information in certain areas. Please explain in more detail how the archetypes were retrieved from BSO and how this alignment was validated.

Thank you for your comment. We would like to clarify that we have only taken the archetypes' classification and the total useful floor area from each archetype from the BSO, the other data being gathered from other data sources. We have clarified these other data sources in detail in lines 381-394.

Although we acknowledge the limitations of the BSO, we still consider it as the most up-to-date framework available in the EU. We have removed the "most advanced" part of the sentence, which indeed might have been praising this framework too much. We nevertheless believe that aligning with the BSO remains relevant, given that it is regularly updated and enriched with new data, making it suitable as part of a long-term data strategy.

Regarding the validation of data, this is indeed a critical component of our analysis. To address this, we have added a dedicated subsection to the methodology that provides a more detailed description of the validation process. You can find the new material in lines 510-534.

For existing buildings, the stock is represented by 66 archetypes per EU MS, resulting in a total of 1,782 archetypes. In addition, we include variants in technical systems, leading to 4,490 archetypes. Each archetype is then assigned information regarding its building geometry, material composition, as well as heating, ventilation and air conditioning systems (HVAC), called attributes. Attributes relative to building geometry are first derived from AmBIENCe[16], then complemented with data from the Cost-effectiveness studies[42] and TABULA/EPISCOPE[43]. Regarding material composition, Hotmaps[17] is used to derive the materials composing floors, walls, roofs and windows, and the construction technologies are then detailed in terms of layer thickness and performance properties using AmBIENCe[16]. In order to validate the retrieved information and fill data gaps, a data review by regional experts who were part of the EU-WLC project[44] was conducted. Finally, all attributes related to HVAC were derived from AmBIENCe[16]. The existing building stock is characterized by its total useful floor area, which is taken from the BSO[18], alongside with building stock occupancy data, for different building archetypes (shown in Figures S1 and S2 in the SI).

4.4. Calibration and validation of the model

The building stock model and its data are both calibrated and validated to ensure representativeness. Calibration is performed for new building construction, thereby directly affecting upfront embodied emissions, and for operational emissions. Together, they represent a large majority of the life cycle emissions of the building stock in the baseline year (2020). In particular, new building constructions are calibrated for the baseline year to reach 80% of the delivered building permits[61] averaged between 2014 and 2023 as a reference value. This is meant to consider a 20% difference between the number of building permits and the actual number of buildings built (as not all permits are actually built). Regarding operational emissions (B6) a calibration for the baseline year (2020) is also performed, using reference emissions for 2020 from the BSO[18]. Direct emissions are overtaken as provided and, due to differences in modelling scope, only indirect emissions for HVAC are considered (other electricity uses are not included).

Validation of the model is performed in two steps. First, the archetype-related attributes underwent multiple validation rounds by regional experts who were part of the EU-WLC project[44], as mentioned in Sections 4.1 and 4.2. Additionally, we use an existing, peer-reviewed model to critically review and verify the validity of the modelling results obtained from the main modelling pipeline, for the baseline year and the BAU scenario. The established MESSAGEix-Buildings model, a bottom-up building sector model to assess energy, material demands, and GHG emissions of buildings at the regional and global scales under different socioeconomic, technological, climate and policy scenarios, as well as the STURM model, a stock turnover model based on dynamic material flow analysis to assess new constructions, demolitions and renovation activities, are deployed for generating those additional validation runs[21,22], as shown in the SI (Figures S7 and S8). The comparison demonstrates that the PULSE-EU produces results within a reasonable and credible range when benchmarked against an independently developed, peer-reviewed framework. Minor discrepancies can be attributed to differences in data sources, modeling assumptions, and structural formulations between the two models.

The choice of using a 50-year reference study period for assessing archetypes should be justified. Furthermore, clarification is required for the case of existing buildings constructed in the 1970s, 1980s, and 1990s. How was this applied, given that the analysis is reported for 2030, 2040, and 2050?

Thank you for giving us the chance to clarify this modelling choice. We selected a 50-year reference study period for the building archetypes because it aligns with the recommendations of the Level(s) framework and is required under the latest version of the Energy Performance of Buildings Directive. Regarding your second point, we annualize the in-use emissions calculated over those 50 years and assume that, at the stock level, these emissions continue beyond the reference period as long as the buildings remain in service. Thus, a building constructed in the 1970s would still generate in-use emissions in 2040, even though that exceeds its nominal 50-year horizon. We have now made this explanation explicit in the manuscript (see lines 443-449).

The building archetypes are assessed using a reference study period of 50 years, consistent with EU standards[27]. This period applies exclusively to the in-use emissions (B2, B4, B6). However, at the stock level, the service lives of the buildings are assessed differently, as explained in Section 4.3, and it is common to have older than 50 years buildings still in use. To facilitate the upscaling to the building stock level, these in-use emissions are then annualized. These annual emissions are assumed to continuously happen as long as the building is in use, even beyond the 50 years reference period.

The PULSE EU model was originally developed for Austria. Please clarify how this model was adapted to other Member States, and what modifications or assumptions were introduced.

Thank you for your insightful comment. Indeed, the PULSE model was originally developed for Austria. We would like to highlight that, although its initial focus was Austria, the model was designed from the outset to be transparent and adaptable for implementation in other countries. In response to your suggestion, we have now clarified how the PULSE model has been extended to cover the broader EU. These revisions can be found in lines 469-480 of the manuscript.

This model is specifically developed to model the EU building stock, by expanding the foundational PULSE-AT model[59], originally developed for Austria, thereby extending its scope to cover all 27 EU MS. PULSE-EU projects future building stock activities, including new construction, maintenance, renovation, replacement and demolition, following the equations

and upscaling the logic from the Austrian model, with these main adaptations: (i) Austrian-specific data is replaced with EU-specific data. An overview of the input data and sources is available on the GitHub publication of the model (see data availability statement). (ii) The model is adapted to import files generated with the SLiCE[20] building life cycle modelling structure, which affected the upscaling of in-use emissions (B2, B4, B6), as explained in Section 4.2. (iii) A new scenario modelling framework is created, which is explained in Section 4.5. (iv) The upscaling is performed based on useful floor area to align with the latest EPBD requirements[27], which is slightly different from the net floor area used in the Austrian model.

Was the Level(s) framework considered in your study? If so, please comment on how it relates to your methodology. If not, please explain why it was excluded.

Thank you for raising this important point. The Level(s) framework was not explicitly considered in this study, as it was beyond the defined research scope. However, our methodology aligns with several key aspects of the Level(s) framework, particularly Indicator 1.2 (Global Warming Potential). Specifically, our approach incorporates the following elements consistent with Level(s):

- We included Global Warming Potential as one of the primary metrics in our assessment.
- We applied EN15804+A2:2019, distinguishing between GWP–fossil, GWP–biogenic, and GWP–land use and land use change.
- We used m² of useful floor area over a 50-year reference study period, in line with Level(s) recommendations.
- Additionally, our study aligns with Level(s) Indicator 1.1 (Use Stage Energy Performance) by applying Nearly Zero-Energy Building (NZEB) requirements to new archetypes in one of the three variants.

While we did not formally integrate the Level(s) framework, we ensured that our methodology reflected its core principles where relevant. We appreciate the opportunity to clarify this alignment and are open to further discussion if needed.

Please clarify whether the defined archetypes are accessible to the public or research community, and in what format they can be obtained.

Thank you for giving us the chance to clarify this point. We are currently preparing a dedicated data publication for the specified archetypes. In the meantime, you may reach out directly to the corresponding authors of the paper, who will be happy to share the information upon a reasonable request. This detail is also noted in the “Data and Code Availability” statement (see 586-587).

Data regarding the archetypes, from their characteristics to their LCA results, are being prepared for open-access publication. The corresponding authors can be contacted for further information until publication

Comments from Review 3

The manuscript investigates the necessary GHG emission reduction in the building stock of the European Union Member States to achieve the collective GHG emissions target by 2030 and beyond. The topic is of interest, and the research has potential. However, several underlying data and methodological assumptions are missing, making it challenging to understand the results. Moreover,

the discussion is too broad and does not provide actionable insights and tailored takeaways based on the results.

Thank you for your insightful comments. We have incorporated all of your suggestions, clearly detailing the data sources and methodological assumptions, narrowing the discussion, and adding concrete, policy-relevant takeaways. We trust that these revisions have strengthened the manuscript and improved its clarity and impact.

A list of policies and measures considered under each strategy, along with their potential embodied and operational carbon savings, is missing. Are these measures based on national plans submitted to the European Commission, or are they assumed based on the target?

Thank you for the opportunity to clarify our scenario modelling. Rather than activating the strategies after the policy targets are reached, we first activate the strategies to achieve those targets. We have now made this point explicit in the Methods section (lines 580-584).

To address the missing information, we have added the strategies considered for each policy target in Table S7 of the supplementary material. The EU policy targets used for the scenario narratives were identified through a review of the principal EU policy documents. Country-specific policies and GHG-emission-reduction targets were not incorporated, which we recognize as a limitation of this approach; this limitation is now discussed in lines 566-568.

The potential embodied and operational carbon savings associated with each strategy are shown in Figure 3 of the main text and detailed in Table S8. Additionally, the complete list of strategies and their corresponding data are publicly available via the GitHub repository linked in the Data Availability statement.

Finally, we have inserted the following paragraph into the Supplementary Information to further clarify the modelling approach:

When generating the scenarios, especially the HOPE scenario, the relevant strategies to reach these policy targets were gradually activated until the targets were reached. The implementation of each strategy in the policy scenarios is as follows:

- **BAU**: No strategy implemented (targets not reached).
- **HOPE**: Strategies 5 and 9 activated to the maximum level (high for all) for all MS, strategies 6, 7, 8 and 10 activated to half the capacity of each MS. All targets are reached.
- **COPE**: Strategies 5 and 9 activated to the maximum capacity of each MS (low, medium or high), strategies 6, 7, 8 and 10 activated to half the capacity of each MS. Targets are partially reached (only EPBD, RED and WFD).
- **SMART**: All ten strategies are activated to the maximum capacity of each MS (low, medium or high). Targets are implicitly reached (e.g., the emissions in 2030 for which the targets are specified are lower than in the HOPE scenario).

The quantitative data used for the modelling of these strategies, including diffusion rates and GHG emission reduction potentials, are available on GitHub publication of the model.

Figure 3 presents the impact of assumed strategies, but the specific measures are not clear. For example, what does 'implementation of improved materials' mean? Although more information is provided in the supplementary material (Table S2), it is still not clear enough.

Thank you for your comment. We have renamed the strategies to make their titles more explicit, and we have substantially revised the Methods section (particularly lines 537-551) to provide additional detail on the specific measures involved. For readers seeking quantitative information on the strategies, we now direct them to the GitHub repository accompanying the model, where all data used in the analysis are transparently reported.

The modelling of future scenarios in this study is based on the combination of different settings for the implementation of GHG emission reduction strategies, meaning that it relies on the implementation of specific strategies to reduce the whole life GHG emissions of EU buildings. These strategies have been identified based on literature review and stakeholder consultation[14], and further grouped into six main categories: (1) the implementation of circularity measures (including the extension of the service life of buildings through renovation, vacancy reduction, efficient structural material use, as well as increased reuse and recycling), (2) the reduction of per capita space demand (by increasing the use intensity of residential buildings), (3) the shift to low carbon, bio-based materials (through timber construction and bio-based insulation), (4) the reduction of operational emissions (including thermal renovation, exchange in HVAC systems, increased share of renewable energy in the electricity and district heating, as well as the reduction in temperature setpoints), (5) the improvement of material production processes (through efficiency improvements, the use of alternative fuels and innovative technologies in material production, such as carbon capture and storage) and (6) the improvement of transport and construction processes (including alternative fuels use, machine optimization and reduction in transport distances).

The building renovation rates and depths assumed for each scenario are unclear. You mention light, medium, and deep levels, but it is not clear how each level is included in each scenario. The HOPE scenario assumes an accelerated renovation wave, but what does this mean? Is it related to the Renovation Wave strategy goals? How many buildings (or floor area) are renovated by 2030, 2040, and 2050 in each scenario?

Thank you for your comment. We have expanded the description of the "renovation wave" used in the HOPE scenarios and included the quantitative renovation rates directly in the manuscript (see lines 120-125). These rates represent the total renovation rate across the EU, encompassing light, medium, and deep renovations.

For readers interested in the breakdown of light, medium, and deep renovations, the detailed numbers are available in the extended strategy dataset hosted in the GitHub repository linked in the Data Availability statement.

In addition, we have added a table (Table S9) to the Supplementary Information that reports the floor-area totals for buildings constructed, demolished, and renovated under each scenario.

The HOPE scenario models an accelerated renovation wave, to strictly fulfill energy efficiency policy targets (Table S7) peaking at 3.6% in 2030 and staying above 3% until 2040, which results in a rapid decline in operational emissions. In contrast, the COPE scenario, which incorporates plausible implementation constraints, for which the list of criteria is provided in the supplementary information (Table S5), projects a delayed peak in renovation activity to 3% in 2040, while only reaching 2% in 2030.

Which implementation constraints are considered in the COPE scenario, and how are they addressed in the SMART scenarios?

Thank you for your comment. The COPE and SMART scenarios share identical implementation constraints, as illustrated in Figure S6 and listed in Table S5. However, the sets of strategies activated in each scenario differ; this distinction is shown in Table 1 and in the newly added text within the Supplementary Information (excerpted below).

The implementation of each strategy in the policy scenarios is as follows:

- **BAU**: No strategy implemented (targets not reached).
- **HOPE**: Strategies 5 and 9 activated to the maximum level (high for all) for all MS, strategies 6, 7, 8 and 10 activated to half the capacity of each MS. All targets are reached.
- **COPE**: Strategies 5 and 9 activated to the maximum capacity of each MS (low, medium or high), strategies 6, 7, 8 and 10 activated to half the capacity of each MS. Targets are partially reached (only EPBD, RED and WFD).
- **SMART**: All ten strategies are activated to the maximum capacity of each MS (low, medium or high). Targets are implicitly reached (e.g., the emissions in 2030 for which the targets are specified are lower than in the HOPE scenario).

Are country-specific targets and measures to decarbonize the building stock and achieve EU-wide goals considered in the analysis?

Thank you for your comment. We acknowledge that country-specific policies and GHG emission reduction targets were not incorporated, which constitutes a limitation of our approach. Concerning the EU targets, we compared progress toward these goals against aggregated EU emissions and energy consumption rather than evaluating each MS individually. This methodology permits certain MS to achieve reductions more rapidly (when they have greater capacity) while still ensuring that the collective EU target is met. We have added a clarification to the manuscript (lines 580-584).

When generating the scenarios, especially the HOPE scenario, the relevant strategies to reach these policy targets were gradually activated until the targets were reached. The total GHG emissions or energy consumption of the EU were compared to these EU policy targets, but not the ones from the MS. This allows, for example, for MS with higher capacity to reduce emissions faster than others, while still ensuring the overall EU target is achieved.

Besides 'reduce space demand per capita' which other sufficiency measures are considered in the analysis?

Thank you for this comment, which prompted us to clarify the modelled strategies. As Reviewer 1 also noted, the term "sufficiency" was overly vague. We have therefore renamed the strategy to "reduction of per capita space demand." Although this is the sole measure addressed in this particular strategy, other sufficiency measures are included in the remaining strategy packages, for example, vacancy reduction in the circularity package and lower temperature set-points in the operational-emissions reduction package. We have updated lines 537-551 to reflect these changes more clearly.

The modelling of future scenarios in this study is based on the combination of different settings for the implementation of GHG emission reduction strategies, meaning that it relies on the implementation of specific strategies to reduce the whole life GHG emissions of EU buildings. These strategies have been identified based on literature review and stakeholder consultation[14], and further grouped into six main categories: (1) the implementation of

circularity measures (including the extension of the service life of buildings through renovation, vacancy reduction, efficient structural material use, as well as increased reuse and recycling), (2) the reduction of per capita space demand (by increasing the use intensity of residential buildings), (3) the shift to low carbon, bio-based materials (through timber construction and bio-based insulation), (4) the reduction of operational emissions (including thermal renovation, exchange in HVAC systems, increased share of renewable energy in the electricity and district heating, as well as the reduction in temperature setpoints), (5) the improvement of material production processes (through efficiency improvements, the use of alternative fuels and innovative technologies in material production, such as carbon capture and storage) and (6) the improvement of transport and construction processes (including alternative fuels use, machine optimization and reduction in transport distances).

The discussion remains too general and fails to provide country and strategy -specific insights and examples. Statements such as 'reducing operational emissions through thermal renovation and exchange of heating systems remains the highest-impact lever, and must be systematically complemented by measures that mitigate embodied emissions, including material decarbonization, circular economy principles, and sufficiency' are overly broad and generic, do not offer innovative insights, and makes it difficult to understand it in practice.

Thank you for challenging us to provide more specific insights and concrete examples in the discussion. In response, we have thoroughly revised the section to better connect our findings with practical applications and policy implications. The most substantial changes appear in lines 316-342, although the entire discussion has been enhanced throughout.

Sufficiency-oriented policies should prioritize the optimal use of the existing building stock to avoid upfront embodied GHG emissions associated with new construction, while simultaneously reducing operational emissions through higher occupancy and intensified use of floor space. Effective implementation requires coordinated measures such as establishing vacancy registers and applying vacancy taxes to incentivize the re-entry of under-occupied units into the market[35], streamlining permitting procedures for repurposing and retrofits, and offering density bonuses or zoning concessions to reward adaptive reuse[28]. Other examples include raising awareness, rethinking communication and designing effective financial motivation, such as a combination of different financial incentives that reward downsizing through flat swaps, or the introduction of minimum occupancy rates[36]. Public authorities and developers can further stimulate flexible, resilient design by launching competitions and pilot projects focused on adaptable concepts, and by creating neighborhood-level agencies that advise tenants on downsizing or relocation across life stages.

For countries showing high potential to reduce embodied GHG emissions by using bio-based materials or improving materials production processes, as well as reuse and recycling, such as the Estonia, Romania, Ireland or the Netherlands, policy measures should combine incentives, standards, and market-creation mechanisms. Governments can introduce tax credits or subsidies for the manufacturing and procurement of certified bio-based products, while mandating minimum bio-content thresholds in public-sector contracts. In parallel, maintaining and enhancing the carbon sink capacities in land-based systems that provide these bio-based products is crucial[37]. Updating building codes and material specifications to recognize and reward low-embodied emissions bio-based materials encourages their adoption in construction. In parallel, standards for circularity, such as material passports, product-level carbon footprints, and verified recycled-content labels, facilitate traceability and market confidence[28]. Supporting research-and-development programs that improve durability of bio-based polymers accelerates technological maturity. Finally, implementing extended producer

responsibility schemes, take-back obligations, and incentives for industrial symbiosis creates economic drivers for systematic reuse, recycling, and urban-mining of bio-based and conventional materials, closing material loops and lowering overall embodied emissions[28].

Embodied carbon represents the major share of a building's life cycle carbon in new buildings; In existing EU buildings, the high energy consumption for building operation is still high and therefore generates high operational carbon.

Thank you for your insightful comment. We agree with your suggestion and have expanded the discussion to include a dedicated section on strategies for reducing operational carbon emissions (see lines 269-283).

Reducing operational GHG emissions being the most effective lever to reduce life cycle emissions in the majority of the MS, operational emissions policies should translate the main EU-related policies (EPBD, EED and RED) into ambitious national actions. Key measures include adopting comprehensive National Building Renovation Plans that chart a pathway to a climate-neutral stock by 2050, enforcing Minimum Energy Performance Standards for non-residential buildings, and applying the Zero-Emission Building standard to all new construction while progressively incorporating whole-life carbon limits. Supporting instruments such as Energy Performance Certificates, Renovation Passports, and targeted financial incentives must be widely available[27]. The EED's "Energy Efficiency First" principle should guide planning, investment and permitting decisions, with the public sector leading by renovating at least 3 % of its floor area annually and prioritizing high-performance retrofits. Finally, the RED calls for accelerated deployment of on-site and neighborhood renewable generation, streamlined legal frameworks for energy communities, and the integration of digital demand-side flexibility to optimize renewable use in buildings and districts[28]. These policies must be complemented by measures that mitigate embodied emissions, and allow strategies to be deployed where they are most effective, thereby navigating implementation capacity constraints.

Figure 3 is very difficult to read.

Thank you for your feedback. We have adjusted the x-axis scale so that it now caps at 50 % for strategies that do not exceed this threshold, which makes the majority of the bars easier to see. Additionally, we would like to remind you that the raw data underlying this graph are available in the Supplementary Information (Table S8) to ensure full transparency.

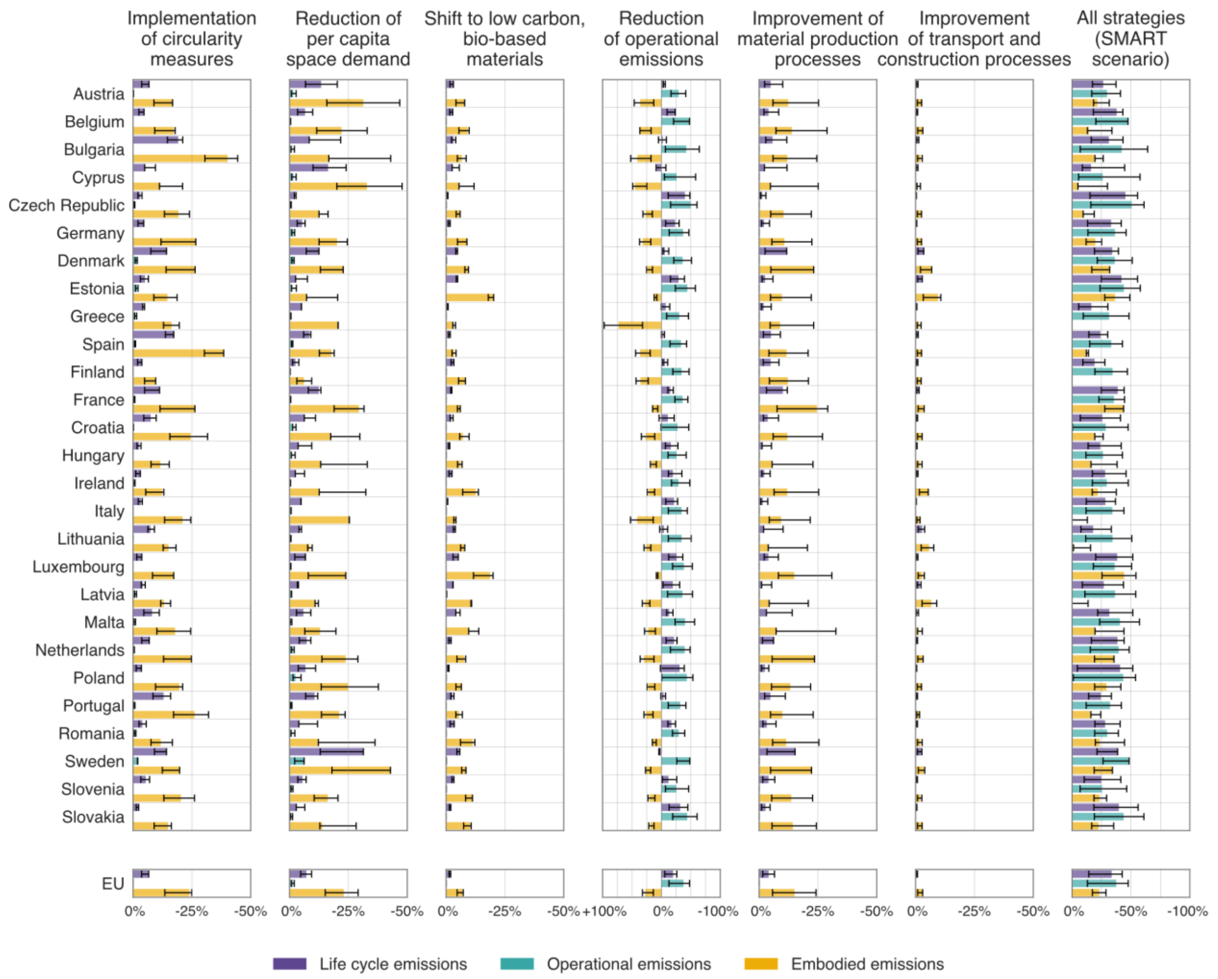

Figure S4, please provide the countries grouping in the four climatic zones.

Thank you for your helpful comment. We have now added a classification of the EU member states into climatic zones, which is included in the Supplementary Information file as Table S2.

What does CRRS stands for in Table S2?

Thank you for pointing that out. "CRRS" actually stands for Carbon Reduction and Removal Strategy. To improve clarity, we have removed the acronym and replaced it in the table with "Strategy number".

NCOMMS-25-61669A: Answer to reviewers

Dear Reviewers,

Thank you for reviewing the paper once more. We appreciate the quality of the review and have revised the paper accordingly. Below is a detailed response to your comments. In bold you will find your original comment, in black indented you will find our response, and in blue indented you will find a copy of the text in the paper that incorporates your comment. The references to specific line numbers refer to the clean version of the article.

Comments from Reviewer 2

All major comments and concerns were adequately addressed.

Thank you for conducting this final review.

Comments from Review 4

In response to Reviewer #3, the manuscript has clearly improved in terms of methodological transparency and the explicit description of strategies, renovation dynamics, and implementation constraints. The clarification of scenario activation logic (HOPE, COPE, SMART), the addition of quantitative renovation rates, and the expanded explanation of strategy categories meaningfully address earlier concerns about missing assumptions. The discussion section has also been strengthened with more concrete examples and country illustrations.

Thank you for recognizing the improvements in the manuscript.

That said, some elements of Reviewer #3's critique are only partially resolved. While strategy effectiveness is differentiated across Member States, the policy implications could benefit from a concise synthesis that explicitly articulates the implied prioritisation emerging from the results (e.g., what generally dominates EU-wide and under which country conditions alternative strategies become more effective).

Many thanks for your comment. We agree that a tighter synthesis would benefit the discussion section and have included it in the first paragraph (lines 192–199).

Across the EU, our results show a clear prioritization: (i) reducing operational emissions is the most effective strategy overall; (ii) reducing per-capita space demand is next-best in MS with high embodied emissions per capita or low-carbon energy mixes; (iii) circularity measures are most effective where operational and embodied emission shares are similar (40–50%); (iv) improvements in material production processes deliver smaller gains on average; and (v) shifting to low-carbon, bio-based materials and improving transport and construction processes typically have the lowest EU-wide impact, albeit with country-specific exceptions (e.g., stronger benefits from bio-based materials in Estonia).

In addition, contextualising the headline mitigation figures (8.53 GtCO_2e and the 2.72 GtCO_2e shortfall) with percentages or benchmarks would improve interpretability.

Thank you for your comment. We have clarified the headline mitigation figures by providing percentages in the abstract (lines 19–22) and main text (lines 92, 94) and by comparing them to annual buildings and construction emissions. We hope this enhances clarity.

Here we show that, over 2020–2050, achieving these targets would require avoiding 8.53 billion metric tons of carbon dioxide equivalent**, approximately ten years of emissions at 2020**

levels. Relying on improving energy efficiency and material production would exceed national capacities by 2.72-billion-ton**, 32 percent of the required reduction**.

A brief expansion on the practical feasibility constraints of sufficiency measures—particularly political and behavioural barriers—would further strengthen credibility.

Thank you for this suggestion. We appreciate this idea and concur that a treatment of sufficiency's practical feasibility was missing. To address it, we added a paragraph in the discussion section, now at lines 256–270.

However, the practical application of sufficiency faces considerable challenges, notably socio-political acceptance and potential rebound effects[36], necessitating fundamental reforms in both the content and the design process of building sector policies[29]. Measures perceived as limiting choice or comfort, such as minimum occupancy thresholds, downsizing incentives, or lower temperature set-points, often face stronger opposition than efficiency standards[35,37]. Instruments like vacancy registers and taxes also require significant administrative capacity and proportional enforcement. Cultural norms around dwelling size, privacy and thermal comfort can limit voluntary uptake or lead to rebound effects. Without careful design and clear exemptions, they may trigger privacy and property-rights concerns or legal challenge. Examples of enablers that could raise acceptability and uptake are comfort guarantees and building-level controls when optimizing temperature set-points, to avoid substitution with portable heaters, or tenant protections and anti-displacement measures when promoting downsizing or intensified use, which could be in the form of temporary rent caps or indexation limits for participants. Evidence additionally indicates that acceptance improves when policies are co-designed with residents and social partners, distribute costs visibly and fairly, preserve choice rather than introduce bans, and deliver clear co-benefits (health, comfort, affordability)[38,39].

Finally, situating the results more explicitly within the current EU policy architecture, including ETS2 and potential financing mechanisms such as ETS2 revenues and the Social Climate Fund, would enhance policy completeness without requiring additional modelling.

Thank you for your insightful comment. It is true that ETS2 and the Social Climate Fund were missing from our discussion, despite their importance in the EU policy architecture. We have thoroughly revised the discussion to include them (see lines 210–229).

Reducing operational GHG emissions being the most effective lever to reduce life cycle emissions in most MS, national policies should translate the EPBD, EED, Renewable Energy Directive (RED), and the forthcoming ETS for buildings and road transport (ETS2) into a coherent package of actions. ETS2 will introduce an upstream carbon price on fossil heating fuels (planned from 2027), which, together with complementary national policies, can reinforce performance standards and targeted support by strengthening incentives for fuel switching and reducing energy demand.

Key additional measures from the EPBD include adopting comprehensive National Building Renovation Plans that chart a pathway to a climate neutral stock by 2050, enforcing Minimum Energy Performance Standards for non-residential buildings, and applying the Zero Emission Building standard to all new construction while progressively incorporating whole life carbon limits. Supporting instruments such as Energy Performance Certificates, Renovation Passports, and targeted financial incentives must be widely available[28]. The EED's Energy Efficiency First principle should guide planning, investment and permitting, with the public sector renovating at least 3 % of its floor area annually and prioritizing deep renovation measures. To ensure

affordability and implementation capacity, MS should earmark a share of ETS2 auction revenues and leverage the Social Climate Fund (2026–2032) to co-finance deep renovations, including exchanges of heating systems, while providing temporary, targeted income support for vulnerable households and micro-enterprises. In parallel, the RED promotes the accelerated deployment of on-site renewable generation and establishes enabling frameworks for energy communities[29]. These policies must be complemented by measures that mitigate embodied emissions, and allow strategies to be deployed where they are most effective, thereby navigating implementation capacity constraints.

Overall, Reviewer #3's main methodological concerns have been substantially addressed, though the clarity and strategic framing of the policy conclusions could still be sharpened.

Thank you for your thoughtful feedback, which has helped us strengthen the manuscript. We have implemented all of your suggestions and hope it now meets the standards for publication.
